# Supplementary material for: Methodological rigor and quality of reporting of clinical trials published with physical activity interventions: A report from the Strengthening the Evidence in Exercise Sciences Initiative (SEES Initiative)
Source: PLoS One. 2024 Aug 30;19(8):e0309087. doi: 10.1371/journal.pone.0309087 (PMC11364220; doi:10.1371/journal.pone.0309087)
Supplement: S1 File — (PDF) [file pone.0309087.s001.pdf]

Supplementary files 1

Supplementary files of article "Methodological rigor and reporting quality of published clinical trials with physical activity interventions: a report of the Strengthening the Evidence in Exercise Sciences Initiative (SEES Initiative)"

Additional file 1- Search strategies

Search strategies for journals of exercise sciences:

MEDLINE/PubMed query for clinical trials:

"Br J Sports Med";[Journal] OR "Med Sci Sports Exerc"[Journal] OR "Eur J Prev Cardiol"[Journal] OR "Sports Med"[Journal] OR "Int J Behav Nutr Phys Act"[Journal] OR "J Sci Med Sport"[Journal] OR "Scand J Med Sci Sports"[Journal] AND ((randomized controlled trial[pt] OR controlled clinical trial[pt] OR randomized controlled trials[mh] OR random allocation[mh] OR double-blind method[mh] OR single-blind method[mh] OR clinical trial[pt] OR clinical trials[mh] OR ("clinical trial"[tw]) OR ((singl*[tw] OR doubl*[tw] OR trebl*[tw] OR tripl*[tw]) AND (mask*[tw] OR blind*[tw])) OR ("latin square"[tw]) OR placebos[mh] OR placebo*[tw] OR random*[tw] OR research design[mh:noexp] OR follow-up studies[mh] OR prospective studies[mh] OR cross-over studies[mh] OR control*[tw] OR prospective*[tw] OR volunteer*[tw]) NOT (animal[mh] NOT human[mh]) NOT (review[ti] OR "meta-analysis"[ti]))

Search strategies for journals in general medicine:

MEDLINE/PubMed query for clinical trials:

"Ann Intern Med"[Journal] OR "BMJ"[Journal] OR "JAMA"[Journal] OR "Lancet"[Journal] NOT ("Lancet Respir Med"[Journal] OR "Lancet Public Health"[Journal] OR "Lancet Psychiatry"[Journal] OR "Lancet Planet Health"[Journal] OR "Lancet Oncol"[Journal] OR "Lancet Neurol"[Journal] OR "Lancet Infect Dis"[Journal] OR "Lancet Haematol"[Journal] OR "Lancet HIV"[Journal] OR "Lancet Glob Health"[Journal] OR "Lancet Gastroenterol Hepatol"[Journal] OR "Lancet Diabetes Endocrinol"[Journal] OR "Lancet Child Adolesc Health"[Journal]) NOT ("Lancet Respir Med"[Journal] OR "Lancet Public Health"[Journal] OR "Lancet Psychiatry"[Journal] OR "Lancet Planet Health"[Journal] OR "Lancet Oncol"[Journal] OR "Lancet Neurol"[Journal] OR "Lancet Infect Dis"[Journal] OR "Lancet Haematol"[Journal] OR "Lancet HIV"[Journal] OR "Lancet Glob Health"[Journal] OR "Lancet Gastroenterol

Hepatol"[Journal] OR "Lancet Diabetes Endocrinol"[Journal] OR "Lancet Child Adolesc Health"[Journal]) OR “N Engl J Med”[Journal] AND (randomized controlled trial[pt] OR controlled clinical trial[pt] OR randomized controlled trials[mh] OR random allocation[mh] OR double-blind method[mh] OR single-blind method[mh] OR clinical trial[pt] OR clinical trials[mh] OR (“clinical trial”[tw]) OR ((singl*[tw] OR doubl*[tw] OR SEES Initiative - Rationale and Methods trebl*[tw] OR tripl*[tw]) AND (mask*[tw] OR blind*[tw])) OR (“latin square”[tw]) OR placebos[mh] OR placebo*[tw] OR random*[tw] OR research design[mh:noexp] OR follow-up studies[mh] OR prospective studies[mh] OR cross-over studies[mh] OR control*[tw] OR prospectiv*[tw] OR volunteer*[tw]) AND (exercise[tiab] OR “physical activity”[tiab] OR training[tiab] OR rehabilitation[tiab]) NOT (animal[mh] NOT human[mh]) NOT (review[ti] OR “meta-analysis”[ti])

Additional File 2- PRISMA CHECKLIST

Section and Topic	Item #	Checklist item	Location where item is reported
**TITLE**			
Title	1	Identify the report as a systematic review.	23
**ABSTRACT**			
Abstract	2	See the PRISMA 2020 for Abstracts checklist.	24
**INTRODUCTION**			
Rationale	3	Describe the rationale for the review in the context of existing knowledge.	27
Objectives	4	Provide an explicit statement of the objective(s) or question(s) the review addresses.	28
**METHODS**			
Eligibility criteria	5	Specify the inclusion and exclusion criteria for the review and how studies were grouped for the syntheses.	29
Information sources	6	Specify all databases, registers, websites, organisations, reference lists and other sources searched or consulted to identify studies. Specify the date when each source was last searched or consulted.	30
Search strategy	7	Present the full search strategies for all databases, registers and websites, including any filters and limits used.	30 MS
Selection process	8	Specify the methods used to decide whether a study met the inclusion criteria of the review, including how many reviewers screened each record and each report retrieved, whether they worked independently, and if applicable, details of automation tools used in the process.	30
Data collection process	9	Specify the methods used to collect data from reports, including how many reviewers collected data from each report, whether they worked independently, any processes for obtaining or confirming data from study investigators, and if applicable, details of automation tools used in the process.	31

Section and Topic	Item #	Checklist item	Location where item is reported
Data items	10a	List and define all outcomes for which data were sought. Specify whether all results that were compatible with each outcome domain in each study were sought (e.g. for all measures, time points, analyses), and if not, the methods used to decide which results to collect.	30
	10b	List and define all other variables for which data were sought (e.g. participant and intervention characteristics, funding sources). Describe any assumptions made about any missing or unclear information.	31
Study risk of bias assessment	11	Specify the methods used to assess risk of bias in the included studies, including details of the tool(s) used, how many reviewers assessed each study and whether they worked independently, and if applicable, details of automation tools used in the process.	NA
Effect measures	12	Specify for each outcome the effect measure(s) (e.g. risk ratio, mean difference) used in the synthesis or presentation of results.	32
Synthesis methods	13a	Describe the processes used to decide which studies were eligible for each synthesis (e.g. tabulating the study intervention characteristics and comparing against the planned groups for each synthesis (item #5)).	29
	13b	Describe any methods required to prepare the data for presentation or synthesis, such as handling of missing summary statistics, or data conversions.	30
	13c	Describe any methods used to tabulate or visually display results of individual studies and syntheses.	31
	13d	Describe any methods used to synthesize results and provide a rationale for the choice(s). If meta-analysis was performed, describe the model(s), method(s) to identify the presence and extent of statistical heterogeneity, and software package(s) used.	NA
	13e	Describe any methods used to explore possible causes of heterogeneity among study results (e.g. subgroup analysis, meta-regression).	NA
	13f	Describe any sensitivity analyses conducted to assess robustness of the synthesized results.	32
Reporting bias assessment	14	Describe any methods used to assess risk of bias due to missing results in a synthesis (arising from reporting biases).	NA
Certainty assessment	15	Describe any methods used to assess certainty (or confidence) in the body of evidence for an outcome.	NA

Section and Topic	Item #	Checklist item	Location where item is reported
**RESULTS**			
Study selection	16a	Describe the results of the search and selection process, from the number of records identified in the search to the number of studies included in the review, ideally using a flow diagram.	34
	16b	Cite studies that might appear to meet the inclusion criteria, but which were excluded, and explain why they were excluded.	34
Study characteristics	17	Cite each included study and present its characteristics.	MS
Risk of bias in studies	18	Present assessments of risk of bias for each included study.	NA
Results of individual studies	19	For all outcomes, present, for each study: (a) summary statistics for each group (where appropriate) and (b) an effect estimate and its precision (e.g. confidence/credible interval), ideally using structured tables or plots.	35
Results of syntheses	20a	For each synthesis, briefly summarise the characteristics and risk of bias among contributing studies.	NA
	20b	Present results of all statistical syntheses conducted. If meta-analysis was done, present for each the summary estimate and its precision (e.g. confidence/credible interval) and measures of statistical heterogeneity. If comparing groups, describe the direction of the effect.	NA
	20c	Present results of all investigations of possible causes of heterogeneity among study results.	NA
	20d	Present results of all sensitivity analyses conducted to assess the robustness of the synthesized results.	41
Reporting biases	21	Present assessments of risk of bias due to missing results (arising from reporting biases) for each synthesis assessed.	NA
Certainty of evidence	22	Present assessments of certainty (or confidence) in the body of evidence for each outcome assessed.	NA
**DISCUSSION**			

Section and Topic	Item #	Checklist item	Location where item is reported
Discussion	23a	Provide a general interpretation of the results in the context of other evidence.	42
	23b	Discuss any limitations of the evidence included in the review.	NA
	23c	Discuss any limitations of the review processes used.	45
	23d	Discuss implications of the results for practice, policy, and future research.	45
**OTHER INFORMATION**			
Registration and protocol	24a	Provide registration information for the review, including register name and registration number, or state that the review was not registered.	NA
	24b	Indicate where the review protocol can be accessed, or state that a protocol was not prepared.	17
	24c	Describe and explain any amendments to information provided at registration or in the protocol.	NA
Support	25	Describe sources of financial or non-financial support for the review, and the role of the funders or sponsors in the review.	47
Competing interests	26	Declare any competing interests of review authors.	47
Availability of data, code and other materials	27	Report which of the following are publicly available and where they can be found: template data collection forms; data extracted from included studies; data used for all analyses; analytic code; any other materials used in the review.	48

From: Page MJ, McKenzie JE, Bossuyt PM, Boutron I, Hoffmann TC, Mulrow CD, et al. The PRISMA 2020 statement: an updated guideline for reporting systematic reviews. BMJ 2021;372:n71. doi: 10.1136/bmj.n71

For more information, visit: http://www.prisma-statement.org/

Additional file 3- individual assessment items

Full assessment of RCTs with individual items, associated component labels (i.e., domains) and, whether applicable, the guideline or tool of reference. The items used to evaluate the RCTs were based on (1) what is expected in studies using physical activity interventions or structured exercise programs and (2) presentation of methodological standards for reproducibility purposes, both in clinical practice and in the scientific field.

Items selected for assessment of RCTs.

Items for assessment by the SEES Initiative	Reference (guideline, checklist item; unless otherwise stated)
Title identification as a randomized trial	CONSORT, 1a
Registration	CONSORT, 23
Protocol	CONSORT, 24
Abstract: interventions intended for each group	CONSORT, Table 2
Abstract: primary outcome	CONSORT, Table 2
Abstract: effects sizes and their precisions (if quantitative results are presented)	CONSORT, Table 2
Abstract: p value	CONSORT 12a
Description of specific objectives, as reported in introduction	CONSORT, 2b
Rationale or goals related to the intervention	TIDIER, 2
Statement of directional hypotheses or an exploratory approach	CONSORT, 2b
Description of trial design	CONSORT, 3a
Eligibility criteria for participants	CONSORT, 4a
Definitions of primary and secondary outcomes	CONSORT, 6a
Description of outcome measurement, as reported in methods	CONSORT, 6a
Mention the use of a reporting guideline	Not applicable
Method and type of randomization	CONSORT, 8a and 8b
Allocation concealment	CONSORT, 9
Blinding/masking for measurements or analysis of outcomes	CONSORT, 11a
Sample size calculation	CONSORT, 7a
Identification of the intervention	TIDieR, 1
Materials used as part of the intervention	TIDieR, 3

Procedures or processes in each ot the interventions	TIDieR, 4
Individuals who were involved in providing the intervention	TIDieR, 5
Modes of delivery of the intervention	TIDieR, 6
Location where the intervention occurred	TIDieR, 7
Period, amount, intensity and schedule of delivery	TIDieR, 8
Description of any individual tailoring for the intervention	TIDieR, 9
Materials/strategies used regarding the intervention adherence	TIDieR, 11
Results of intervention adherence	TIDieR, 12
Statistical methods for group comparison	CONSORT, 12a
Adherence to either the intention-to-treat principle or per-protocol strategy	CONSORT 16
Numbers of participants who were assigned, received intervention, and were analyzed for the primary outcome	CONSORT, 13a
Dates/periods of recruitment	CONSORT, 14a
Baseline data for each group	CONSORT, 15
Actual number of participants analyzed	CONSORT, 16
Effect sizes described with their precision measures	CONSORT, 17a
Participant flow information, as reported in results	CONSORT, 13a
Non-planned changes after the trial commenced	TIDIER, 10
Changes to the the ORIGINAL protocol during the study	CONSORT, 6b
Harm or unintended effects	CONSORT, 19
Discussion of limitations (potential bias, imprecision, etc)	CONSORT, 20
Presence of potential spin bias	Based on Boutron et al (11)
Statement regarding data availability	Based on ICMJE (12)
Statement regarding sources of funding	CONSORT, 25
Statement regarding potential conflicts of interest	Not applicable

ICMJE: International Committee of Medical Journal Editors; CONSORT: Consolidated Standards of Reporting Trials; TIDieR: template for intervention description and replication.

Full assessment of RCT with individual items and associated component labels.

Component label	Number of items
Transparency	5
Completeness	10
Participants	2
Intervention	9
Methodological rigor	5
Outcome	9
Critical appraisal	4

Additional file 4: Exceptions and operationalization of the answers of some items

Items where we considered more than one answer, the answer “No” or another answer as being "positive" (i.e., recommended practice):

1. **Abstract: If any quantitative result is reported, is it accompanied by the respective precision estimate (e.g., standard deviation, confidence intervals, etc)?" (Outcome)**

The possibilities were: “Yes”, “No” or “There is no reporting of quantitative results”. Therefore, the last one was considered “No”.

2. **Abstract: Is a P-value reported in the abstract?" (Outcome)**

The possibilities were: “No”, “Yes, with P value(s) reported at varied levels (BOTH equal/lower AND higher than 0.05)” or “Yes, with P value(s) reported at a level EQUAL or LOWER than 0.05”. Therefore, these two last possibilities were combined as “Yes”, when the result of this question was presented in binary form (i.e., yes or no).

3. **Abstract: Does the abstract inform the primary outcome (variable of interest)?" (Outcome)**

The possibilities were: “No”, “Yes” or “Unclear”. The last one was used when it was not possible to clearly identify the outcomes. This way, “UNCLEAR” was classified as“NO”

4. **Introduction: Is there a hypothesis stated for the outcome(s) of interest? (Critical appraisal)**

The possibilities were: “No”, “Yes”, “Does not apply”, or “Unclear” .When there wasn’t a directional hypothesis, but there is explanation for an exploratory approach, the answer, this option was considered “YES”. Therefore when it was not possible to clearly identify the outcomes, “UNCLEAR” was classified as“NO”.

5. **Methods: Is there a detailed description of eligibility criteria for participants? (i.e., information that would allow to replicate the inclusion and exclusion decisions)" (Participants)**

The possibilities were: “No”, “Yes” or “Unclear”.When it was not possible to clearly identify the eligibility criteria, “UNCLEAR” was classified as“NO”.

6. **Methods: Are primary and secondary outcomes listed?" (Outcomes)**

The possibilities were: “No”, “Yes”, “Partially yes”, or “Unclear”. In this case we assume that, although incomplete, the topic was approached, so “Partially yes”was classified as“YES”. When it was not possible to clearly identify the eligibility criteria, “UNCLEAR” was classified as“NO”.

7. **Methods: Is the type of randomization sufficiently described? (Note: (i) type, such as computer; (ii) allocation ratio; and (iii) methods of restriction (if any) such as stratification or blocking should be considered)" (Methodological rigor)**

The possibilities were: “No”, “Yes” or “Partially yes”. In this case we assume that, although incomplete, the topic was approached, so “Partially yes”was classified as“YES”

8. **Methods-TID3: Is there a description of physical and information materials used as part of the intervention? (i.e., information that would allow to replicate the intervention with materials for participants or intervention providers)" (Intervention)**

   Methods-TID4: Is there a description of activities or procedures used to carry out the intervention? (i.e., information that would allow to replicate the necessary steps to run the intervention)" (Intervention)

The possibilities were: “No”, “Yes” or “Unclear”.When it was not possible to clearly identify the eligibility criteria, “UNCLEAR” was classified as“NO”.

9. **Methods-TID5: Is there a description of individuals involved in providing the intervention? (i.e., information that would allow to replicate the necessary workforce to run the intervention)" (Intervention)**

   Methods-TID6: Is there a description of modes of delivery of the intervention? (i.e., information that would allow to replicate the delivery to run the intervention, such as face-to-face, telefone, individually or in a group)" (Intervention)

Methods/Results-TID8: Is there a description regarding the period, amount, intensity and schedule of delivery? (i.e., information that would allow to replicate 'when' and 'how much')" (Intervention)

The possibilities were: “No”, “Yes” or “Partially yes”. In this case we assume that, although incomplete, the topic was approached, so “Partially yes”was classified as“YES”

10. **Methods/Results-TID9: If any form of individual tailoring for the intervention was used (such as, variable exercise intensity), is there a description for the rationale and guide for tailoring?" (Intervention).**

The possibilities were: “No”, “Yes”, “Does not apply”, or “Unclear” ..When there was no individual directional intervention, but there is an explanation for this, the answer, this option was considered “YES”. Therefore when it was not possible to clearly identify the outcomes, “UNCLEAR” was classified as“NO”.

11. **Methods/Results-TID11: Is there a description of materials/strategies used in regard to intervention adherence?" (Intervention)[69]**

Methods/Results/Discussion: Is there any mention of changes to the ORIGINAL protocol during the course of the study? (Note: regards to ANY study component, including design, subjects, sampling, measurements, interventions, analyses, etc)." (Transparency)

The possibilities were: “No”, “Yes” or “Does not apply”. .When there was no individual directional intervention, but there is an explanation for this, the answer, this option was considered “YES”.

12. **Discussion: Is there a potential spin bias based on a specific reporting strategy to highlight that the experimental treatment is beneficial?"**

The possibilities were: “No” or “Yes”. The assessment is NO that there was no spin bias, however, to facilitate the analysis “No” was considered “Yes”.

Additional file 5. List of the studies in descending order of adherence to the items/practices recommended

1. Randomised controlled trial of an augmented exercise referral scheme using web-based behavioural support for inactive adults with chronic health conditions: the e-coachER trial
   Taylor A , Taylor R, Ingram W, Dean SG, Jolly K, Mutrie N, Lambert J, Yardley L, Streeter A, Greaves C, McAdam C , Price L, Anokye NK, Campbell J. Br J Sports Med. 2020. Apr. 55(8):444-450. doi: 10.1136/bjsports-2020-103121.
   PMID: 33247001
   Free PMC article

2. Optimising outcomes of exercise and corticosteroid injection in patients with subacromial pain (impingement) syndrome: a factorial randomised trial
   Roddy E, Ogollah R O, Oppong R, Zwierska I, Datta P, Hall A, Hay E, Jackson S, Jowett S, Lewis M, Shufflebotham J, Stevenson K, Wind A, Young J, Foster N E. Br J Sports Med. 2020. Mar. 55(5):262-271. doi: 10.1136/bjsports-2019-101268.
   PMID: 32816787
   Free PMC article

3. Efficacy of heel lifts versus calf muscle eccentric exercise for mid-portion Achilles tendinopathy (HEALTHY): a randomised trial
   Rabusin C L, Menz H B, McClelland J A, Evans A M, Landorf KB, Malliaras P, Docking S I, Munteanu S E. BMC Medical. 2020. Mar. 21;12:20. doi: 10.1186/s13047-019-0325-2.
   PMID: 30949243
   Free PMC article

4. Forty-five per cent lower acute injury incidence but 2 effect on overuse injury prevalence in youth floorball players (aged 12–17 years) who used an injury prevention exercise programme: two-armed parallel group cluster randomised controlled trial
   Åkerlund I, Waldén M, Sonesson S, Hägglund M. Br J Sports Med. 2020. Sep;54(17):1028-1035. doi: 10.1136/bjsports-2019-101295.
   PMID: 31992545
   Free PMC article

5. Does foot mobility affect the outcome in the management of patellofemoral pain with foot orthoses versus hip exercises? A randomised clinical trial
Matthews M, Rathleff M S, Claus A, McPoil T, Nee R, Crossley K M, Kasza J, Vicenzino B T. Br J Sports Med. 2020. Dec. 54(23):1416-1422. doi: 10.1136/bjsports-2019-100935.
PMID: 32217524

6. Effectiveness of blood flow-restricted slow walking on mobility in severe multiple sclerosis: A pilot randomized trial
Lamberti N, Straudi S, Donadi M, Tanaka H, Basaglia N, Manfredini F. Scand J Med Sci Sports. 2020 Oct;30(10):1999-2009. doi: 10.1111/sms.13764.
PMID: 32645227

7. A physical activity coaching intervention can improve and maintain physical activity and health-related outcomes in adult ambulatory hospital patients: the Healthy4U-2 randomised controlled trial
Barrett S, Begg S, O'Halloran P, Kingsley M. Int J Behav Nutr Phys Act . 2020 Nov 30;17(1):156. doi: 10.1186/s12966-020-01063-x.
PMID: 33256753
Free PMC article

8. One-year aerobic interval training in outpatients with schizophrenia: A randomized controlled trial
Brobakken M F, Nygård M, Güzey I C, Morken G, Reitan S K, Heggelund J, Kjelsaas E V, Wang E. Scand J Med Sci SportS . 2020 Dec;30(12):2420-2436. doi: 10.1111/sms.13808.
PMID: 33108030

9. Effectiveness of prescribing physical activity in parks to improve health and wellbeing - the park prescription randomized controlled trial
Riemenschneider F M, Petrunoff N, Yao J, Ng A, Sia A, Ramiah A, Wong M, Han J, Tai B C, Uijtdewilligen L.Int J Behav Nutr Phys Act. 2020 Mar; 17(42). doi:10.1186/s12966-020-00941-8.

PMID:32183815
Free PMC article

10. Results of caring and reaching for healt (CARE): a cluster-randomized controlled trial assessing a worksite wellness intervention for child care staff
Linnan LA, Vaughn A E, Smith F T, Westgate P, Hales D, Arandia G, Neshteruk C, Willis E, Ward D S. Int J Behav Nutr Phys Act. 2020 May 15;17(1):64. doi: 10.1186/s12966-020-00968-x
PMID:32414381

11. Scale-up of the Physical Activity 4 Everyone (PA4E1) intervention in secondary schools: 12-month implementation outcomes from a cluster randomized controlled trial
Sutherland R, Campbell E, McLaughlin M, Nathan N, Wolfenden L, Lubans D R, Morgan P J, Gillham K, Oldmeadow C, Searles A, Reeves P,Williams M, Evans N, Bailey A, Boyer J, Lecathelinais C, Davies L, McKenzie T, Robertson K, Wiggers J. Int J Behav Nutr Phys Act 2021 Oct 23;18(1):137. doi: 10.1186/s12966-021-01206-8.
PMID:34688281
Free PMC article

12. Efficacy of the ‘Stand and Move at Work’ multicomponent workplace intervention to reduce sedentary time and improve cardiometabolic risk: a group randomized clinical trial
Pereira M A, Mullane S L, Toledo M J L, Larouche M L, Rydell S A, Vuong B, Feltes L A, Mitchell N R, Brito J N, Hasanaj K, Carlson N G, Gaesser G A, Crespo N C, Oakes J M, Buman M P. Int J Behav Nutr Phys Act. 2020 Oct 27;17(1):133. doi: 10.1186/s12966-020-01033-3.
PMID: 33109190
Free PMC article

13. Time-efficient intervention to improve older adolescents’ cardiorespiratory fitness: findings from the ‘Burn 2 Learn’ cluster randomised controlled trial
Lubans D R, Smith J J, Eather N,Leahy A A, Morgan P J, Lonsdale C, Plotnikoff R C, Nilsson M, Kennedy S G, Holliday E G, Weaver N, Noetel M, Shigeta T T, Mavilidi M

F, Valkenborghs S R, Gyawali P, Walker F R, Costigan S A, Hillman C H. Br J Sports Med. 2020 Dec 21;55(13):751-758. doi: 10.1136/bjsports-2020-103277.
PMID: 33355155
Free PMC article

14. Cardiorespiratory fitness and accelerometer-determined physical activity following one year of free-living high- intensity interval training and moderate- intensity continuous training: a randomized trial
Jung M E, Locke S R, Bourne J E, Beauchamp M R; , Lee T, Singer J, MacPherson M, Barry J, Jones C, Little JP. Int J Behav Nutr Phys Act. 2020 JAN 17(25). doi: 10.1186/s12966-020-00933-8
PMID: 32102667

15. Does an effective shoulder injury prevention program affect risk factors in handball? A randomized controlled study
Fredriksen H, Cools A, Bahr R , Myklebust G. Scand J Med Sci Sports. 2020 Aug;30(8):1423-1433. doi: 10.1111/sms.13674.
PMID:32293738

16. Combination of resistance and aerobic exercise for six months improves bone mass and physical function in HIV infected individuals: A Randomized Controlled Trial
Ghayomzadeh M, Earnest C P, Hackett D, SeyedAlinaghi S, Navalta J W, Gholami M, Rouzbahani N H, Mohraz M, Voltarelli F Z. Scand J Med Sci Sports. 2021 Mar;31(3):720-732. doi: 10.1111/sms.13871
PMID: 33185897

17. Effects of Land versus Water Walking Interventions on Vascular Function in Older Adults
Haynes A, Naylor L H, Spence L A, Robey E, Cox K L, Maslen B A, Lautenschlager N T, Carter H H, Ainslie P N, Green D J. Med Sci Sports Exerc. 2021 Jan;53(1):83-89. doi: 10.1249/MSS.0000000000002439.
PMID: 32555027

18. The effect of a school-based intervention on physical activity, cardiorespiratory fitness and muscle strength: the School in Motion cluster randomized trial
Kolle E, Solberg R B, Säfvenbom R , Dyrstad S M , Berntsen S, Resaland G K, Ekelund U, Anderssen S A, Johannessen J S, Grydeland M.Int J Behav Nutr Phys

Act. 2020 Nov 26;17(1):154. doi: 10.1186/s12966-020-01060-0.
PMID: 33243246
Free PMC article

19. Does load management using the acute:chronic workload ratio prevent health problems? A cluster randomised trial of 482 elite youth footballers of both sexes
Lorentsen T D, Bjørneboe J, Clarsen B, Vagle M, Fagerland M W, Andersen T E. Br J Sports Med. 2021 Jan;55(2):108-114. doi: 10.1136/bjsports-2020-103003.
PMID: 33036995

20. Combined aerobic/resistance/inspiratory muscle training as the 'optimum' exercise programme for patients with chronic heart failure: ARISTOS-HF randomized clinical trial
Laoutaris L D, Piotrowicz E, Kallistratos M S, Dritsas A, Dimaki N, Miliopoulos D, Andriopoulou M, Manolis A J, Volterrani M, Piepoli M F,Coats A J S, Adamopoulos S. Eur J Prev Cardiol. 2021 Dec 29;28(15):1626-1635. doi: 10.1093/eurjpc/zwaa091
PMID: 33624071

21. Feasibility and Effects on Muscle Function of an Exercise Program for Older Adults
Minett M M, Binkley T L, Holm R P, Runge M, Specker B L. Med Sci Sports Exerc. 2020 Feb;52(2):441-448. doi: 10.1249/MSS.0000000000002152.
PMID: 31939912

22. Effect of Functional Impact Training on Body Composition, Bone Mineral Density, and Strength in Breast Cancer Survivors
Artese A L, Hunt R L, Ormsbee M J, Kim J, Arjmandi B H, Panton L B. Med Sci Sports Exerc. 2021 Jan;53(1):90-101. doi: 10.1249/MSS.0000000000002438.
PMID:32694366

23. Acute Running and Coronary Heart Disease Risk Markers in Male Cigarette Smokers and Nonsmokers: A Randomized Crossover Trial
Alotaibi T F, Thackray A E, Roberts M J, Alanazi T M, Bishop N C, Wadley A J, King J A, O'Donnell E, Steiner M C, Singh S J, Stensel DJ. Med Sci Sports Exerc. 2021 May 1;53(5):1021-1032. doi: 10.1249/MSS.0000000000002560.
PMID:33196606
Free PMC article

24. The Coronary ARteriogenesis with combined Heparin and EXercise therapy in chronic refractory Angina (CARHEXA) trial: A double-blind, randomized, placebo-controlled stress echocardiographic study
Petrovic M T, Dikic A D, Giga V, Boskovic N, Vukcevic V, Cvetic V, Mladenovic A, Radmili O, Markovic Z, Dobric M, Aleksandric S, Tesic M, Juricic S, Beleslin B N, Stojkovic S, Ostojic M C, Beleslin B, Picano E.Eur J Prev Cardiol. 2020 Apr 9;2047487320915661. doi: 10.1177/2047487320915661
PMID:33611455

25. Using TENS to Enhance Therapeutic Exercise in Individuals with Knee Osteoarthritis
Pietrosimone B, Luc-Harkey B A, Harkey M S, Davis-Wilson H C, Pfeiffer S J, Schwartz T A, Nissman D, Padua D A, Blackburn J T, Spang J T. Med Sci Sports Exerc. 2020 Oct;52(10):2086-2095. doi: 10.1249/MSS.0000000000002353.
PMID: 32251254

26. Prenatal Exercise and Cardiovascular Health (PEACH) Study: Impact on Muscle Sympathetic Nerve (Re)activity
Skow R J, Fraser G M, Steinback C D, Davenport M H. Med Sci Sports Exerc. 2021 Jun 1;53(6):1101-1113. doi: 10.1249/MSS.0000000000002583.
PMID: 33315812

27. Effects of Exercise Mode on Postprandial Metabolism in Humans with Chronic Paraplegia
McMillan D W, Maher J L, Jacobs K A, Mendez A J, Nash M S, Bilzon J L. Med Sci Sports Exerc. 2021 Jul 1;53(7):1495-1504. doi: 10.1249/MSS.0000000000002593.
PMID: 33433151

28. Effects of the FIFA 11+ on injury prevention in amateur futsal players
Lopes M, Simões D, Costa R, Oliveira J, Ribeiro F. Scand J Med Sci Sports. 2020 Aug;30(8):1434-1441. doi: 10.1111/sms.13677
PMID: 32279363

29. Nonexercise Equations for Determining Change in Cardiorespiratory Fitness
Lannoy L D, Ross R. Med Sci Sports Exerc. 2020 Jul;52(7):1525-1531. doi:

10.1249/MSS.0000000000002284.
PMID:31977632

30. Inflammation Mediates Exercise Effects on Fatigue in Patients with Breast Cancer
Hiensch A E, Mijwel S, Bargiela D, Wengström Y, May A M, Rundqvist H. Med Sci Sports Exerc. 2021 Mar 1;53(3):496-504. doi: 10.1249/MSS.0000000000002490.
PMID: 32910094
Free PMC article

31. Patterns and predictors of exercise behavior during 24 months of follow-up after a supervised exercise program during breast cancer chemotherapy
An K, Kang D, Morielli A R, Friedenreich C M, Reid R D, McKenzie D C, Gelmon K, Mackey J R, Courneya K S. Int J Behav Nutr Phys Act. 2020 Feb 14;17(1):23. doi: 10.1186/s12966-020-00924-9.
PMID: 32059728
Free PMC article

32. A comparison of acute glycaemic responses to accumulated or single bout walking exercise in apparently healthy, insufficiently active adults
Shambrook P, Kingsley M I, Taylor N F, Wundersitz D W, Wundersitz C E, Paton C D, Gordon B A. J Sci Med Sport 2020 Oct;23(10):902-907. doi: 10.1016/j.jsams.2020.02.015.
PMID: 32173259

33. Exercise and Protein Effects on Strength and Function with Weight Loss in Older Women
Evans E M, Straight C R, Reed R A, Berg A C, Rowe D A, Johnson M A. Med Sci Sports Exerc. 2021 Jan;53(1):183-191. doi: 10.1249/MSS.0000000000002429.
PMID: 32520876

34. Gait biofeedback and impairment-based rehabilitation for chronic ankle instability
Koldenhoven R M, Jaffri A H, DeJong A F, Abel M, Hart J, Saliba S, Hertel J. Scand J Med Sci Sports. 2021 Jan;31(1):193-204. doi: 10.1111/sms.13823.
PMID: 32939858

35. Both Traditional and Stair Climbing-based HIIT Cardiac Rehabilitation Induce Beneficial Muscle Adaptations

Lim C, Dunford E C, Valentino C E, Oikawa S Y, McGlory C, Baker S K, Macdonald M J, Phillips S M. Med Sci Sports Exerc. 2021 Jun 1;53(6):1114-1124. doi: 10.1249/MSS.0000000000002573.
PMID: 33394901

36. Impact of low-volume concurrent strength training distribution on muscular adaptation
Kilen A, Bay J, Bejder J, Andersen A B, Bonne T C, Larsen P D, Carlsen A, Egelund J, Nybo L, Mackey A L, Olsen N V, Aachmann-Andersen N J, Andersen J L, Nordsborg N B. J Sci Med Sport. 2020 Oct;23(10):999-1004. doi: 10.1016/j.jsams.2020.03.013.
PMID: 32371120.

37. The causal pathway effects of a physical activity intervention on adiposity in children: The KISS Study cluster randomized clinical trial
Lima R A, Andersen L B, Soares F C, Kriemler S. Scand J Med Sci Sports. 2020 Jun, 30(9): 1685-1691. doi:10.1111/sms.13741
PMID:32501613

38. Muscle contractile properties of cancer patients receiving chemotherapy: Assessment of feasibility and exercise effects
Buffart L B, Sweegers M G, Ruijter C J, Konings I R, Verheul R M W, van Zweeden A A, Grootscholten C, Chinapaw M J, Altenburg T M. Scand J Med Sci Sports. 2020 Jun, 30(10):1918-1929. doi: 10.1111/sms.13758.
PMID:32599670

39. Endurance and Sprint Training Improve Glycemia and VO2peak, but only Frequent Endurance Benefits Blood Pressure and Lipidemia
Petrick H L, King T J, Pignanelli C, Vanderlinde T E, Cohen J N, Holloway G P, Burr J F. Med Sci Sports Exerc. 2021 Jun 1;53(6):1194-1205. doi: 10.1249/MSS.0000000000002582.
PMID: 33315809

40. The “11 for Health in Denmark” intervention in 10- to 12-year-old Danish girls and boys and its effects on well-being—A large-scale cluster RCT
Madsen M, Elbe A, Madsen E E, Ermidis G, Ryom K, Wikman J M, Lind R R, Larsen M N, Krustrup P. Scand J Med Sci Sports. 2020 Sep;30(9):1787-1795. doi: 10.1111/sms.13704.

PMID: 32353906

41. Multidirectional Walking in Hematopoietic Stem Cell Transplant Patients
Potiaumpai M, Cutrono S, Medina T, Koeppel M, Pereira D L, Pirl W F, Jacobs K A, Eltoukhy M, Signorile J F. Med Sci Sports Exerc. 2021 Feb 1;53(2):258-266. doi: 10.1249/MSS.0000000000002474.
PMID: 32735114

42. 'Maths on the move': Effectiveness of physically-active lessons for learning maths and increasing physical activity in primary school students
Vetter M, O'Connor H T, O'Dwyer N, Chau J, Orr R. J Sci Med Sport. 2020 Aug;23(8):735-739. doi: 10.1016/j.jsams.2019.12.019.
PMID: 31926869

43. Blood Flow Restriction Training Blunts Chronic Kidney Disease Progression in Humans
Corrêa H L, Neves R V P, Deus L A, Souza M K, Haro A S, Costa F, Silva V L, Santos C A R, Moraes M R, Simões H G, Navalta J W, Prestes J, Rosa T S. Med Sci Sports Exerc. 2021 Feb 1;53(2):249-257. doi: 10.1249/MSS.0000000000002465.
PMID: 32826635

44. Distribution of concurrent training sessions does not impact endurance adaptation
Kilen A, Bay J, Bejder J, Andersen A B, Bonne T, Larsen P, Carlsen A, Egelund J, Nybo L, Olsen N V, Aachmann-Andersen N J, Andersen J L, Nordsborg N B. J Sci Med Sport.2021 Mar;24(3):291-296. doi: 10.1016/j.jsams.2020.09.009.
PMID: 32998848

45. Effect of Strength Training on Glycemic Control and Adiponectin in Diabetic Children
Petschnig R, Wagner T, Robubi A, Baron R. Med Sci Sports Exerc. 2020 Oct;52(10):2172-2178. doi: 10.1249/MSS.0000000000002356.
PMID: 32301853

46. Reporting of Resistance Training Dose, Adherence, and Tolerance in Exercise Oncology
Fairman C M, Nilsen T S, Newton R U, Taaffe D R, Spry N, Joseph D, Chambers S K, Robinson Z P, Hart N H, Zourdos M C, Focht B C, Peddle-McIntyre C J, Galvão D A. Med Sci Sports Exerc. 2020 Feb;52(2):315-322. doi:

10.1249/MSS.0000000000002127.
PMID: 31436734

47. Training load but not fatigue affects cross-education of maximal voluntary force
Colomer-Poveda D, Romero-Arenas S, Fariñas J, Iglesias-Soler E, Hortobágyi T, Márquez G. Scand J Med Sci Sports. 2021 Feb;31(2):313-324. doi: 10.1111/sms.13844.
PMID: 33038018

48. Effects of velocity loss in the bench press exercise on strength gains, neuromuscular adaptations, and muscle hypertrophy
Pareja-Blanco F, Alcazar J, Cornejo-Daza P J, Sánchez-Valdepeñas J, Rodriguez-Lopez C, Mora J H, Sánchez-Moreno M, Bachero-Mena B, Alegre L M,Ortega-Becerra M. Scand J Med Sci Sports. 2020 Nov;30(11):2154-2166.
PMID: 32681665

49. Velocity Loss as a Critical Variable Determining the Adaptations to Strength Training
Pareja-Blanco F, Alcazar J, Sánchez-Valdepeñas J, Cornejo-Daza P J, Piqueras-Sanchiz F, Mora-Vela R, Sánchez-Moreno M, Bachero-Mena B, Ortega-Becerra M, Alegre L M. Med Sci Sports Exerc. 2020 Aug;52(8):1752-1762. doi: 10.1249/MSS.0000000000002295.
PMID: 32049887

50. Inter-individual variation in response to resistance training in cardiometabolic health indicators
Ahtiainen J P, Sallinen J, Häkkinen K, Sillanpää E. Scand J Med Sci Sports. 2020 Jun;30(6):1040-1053. doi: 10.1111/sms.13650.
PMID: 32150772

51. Acute metabolic responses after continuous or interval exercise in post-menopausal women with overweight or obesity
Dupuit M, Boscaro A, Bonnet A, Bouillon P, Bruno P, Morel C, Rance M, Boisseau N. Scand J Med Sci Sports. 2020 Dec;30(12):2352-2363. doi: 10.1111/sms.13814
PMID: 32881054
Free PMC article

52. Hamstring strength and architectural adaptations following inertial flywheel resistance training

Preslanda J, Opar D, D.Williams M, Hickey J, Maniar N, Dow C, Bourne M, Timmins R. Journal of Science and Medicine in Sport. 2020 Nov; 23(11):1093-1099. doi:10.1016/j.jsams.2020.04.007
PMID:32461050

53. Proprioceptive neuromuscular facilitation improves pain and descending mechanics among elderly with knee osteoarthritis
Song Q, Shen P, Mao M, Sun W, Zhang C, Li L. Scand J Med Sci Sports. 2020 Sep;30(9):1655-1663. doi: 10.1111/sms.13709.
PMID: 32407583
