# Supplementary material for: Methodological rigor and quality of reporting of clinical trials published with physical activity interventions: A report from the Strengthening the Evidence in Exercise Sciences Initiative (SEES Initiative)
Source: PLoS One. 2024 Aug 30;19(8):e0309087. doi: 10.1371/journal.pone.0309087 (PMC11364220; doi:10.1371/journal.pone.0309087)
Supplement: S2 File — (PDF) [file pone.0309087.s002.pdf]

Supplementary file 2

Screening of articles after duplicates removal

PACKS-RCT	PMID	Title	Journal/Book	Excluded or Included	Exclusion reason	Randomized ("YES" if selected)
PACK1	30554146	Physical activity less than the recommended amount may prevent the onset of major biological risk factors for cardiovascular disease: a cohort study of 198 919 adults	Br J Sports Med	Excluded	Non RCT	
PACK1	30890535	AMSSM position statement update: blood-borne pathogens in the context of sports participation	Br J Sports Med	Excluded	Non RCT	
PACK1	30988018	Doubles trouble-85 cases of ocular trauma in badminton: clinical features and prevention	Br J Sports Med	Excluded	Non RCT	
PACK1	31000565	Therapeutic Use Exemptions (TUEs) are essential in sport: but there is room for improvement	Br J Sports Med	Excluded	Non RCT	
PACK1	31023857	To strive is human, to abuse malign: discrimination and non-accidental violence of professional athletes without employee-style	Br J Sports Med	Excluded	Non RCT	

PACKS-RCT	PMID	Title	Journal/Book	Excluded or Included	Exclusion reason	Randomized ("YES" if selected)
		statutory protection				
PACK1	31186258	Wild goose chase - no predictable patient subgroups benefit from meniscal surgery: patient-reported outcomes of 641 patients 1 year after surgery	Br J Sports Med	Excluded	Non RCT	
PACK1	31203198	Compliance with needle-use declarations at two Olympic Winter Games: Sochi (2014) and PyeongChang (2018)	Br J Sports Med	Excluded	Non RCT	
PACK1	31227490	Infographic. Therapeutic exercise relieves pain and does not harm knee cartilage nor trigger inflammation	Br J Sports Med	Excluded	Non RCT	
PACK1	31315826	The Italian preparticipation evaluation programme: diagnostic yield, rate of disqualification and cost analysis	Br J Sports Med	Excluded	Non RCT	
PACK1	31318714	Quantification of Agility Testing with Inertial Sensors after a Knee Injury	Med Sci Sports Exerc	Excluded	Non RCT	

PACKS-RCT	PMID	Title	Journal/Book	Excluded or Included	Exclusion reason	Randomized ("YES" if selected)
PACK1	31399428	Similar risk of ACL graft revision for alpine skiers, football and handball players: the graft revision rate is influenced by age and graft choice	Br J Sports Med	Excluded	Non RCT	
PACK1	31410887	A before-school physical activity intervention to improve cognitive parameters in children: The Active-Start study	Scand J Med Sci Sports	Included		
PACK1	31436734	Reporting of Resistance Training Dose, Adherence, and Tolerance in Exercise Oncology	Med Sci Sports Exerc	Included		YES
PACK1	31506902	RugbySmart: Challenges and Lessons from the Implementation of a Nationwide Sports Injury Prevention Partnership Programme	Sports Med	Excluded	Non RCT	
PACK1	31537549	Preventing catastrophic injury and death in collegiate athletes: interassociation recommendations endorsed by 13 medical and sports medicine organisations	Br J Sports Med	Excluded	Non RCT	

PACKS-RCT	PMID	Title	Journal/Book	Excluded or Included	Exclusion reason	Randomized ("YES" if selected)
PACK1	31581345	Association between physical activity and all-cause mortality: A 15-year follow-up using a compositional data analysis	Scand J Med Sci Sports	Excluded	Non RCT	
PACK1	31584703	Head impact exposure in youth football-Are current interventions hitting the target?	Scand J Med Sci Sports	Excluded	Non RCT	
PACK1	31605551	By failing to prepare, you are preparing your anterior cruciate ligament to fail	Scand J Med Sci Sports	Excluded	Non RCT	
PACK1	31628097	Return-to-sport testing following ACL reconstruction revisited	Br J Sports Med	Excluded	Non RCT	
PACK1	31704696	18 highlights from the International Criteria for ECG interpretation in athletes	Br J Sports Med	Excluded	Non RCT	
PACK1	31796464	How do the new Olympic sports compare with the traditional Olympic sports? Injury and illness at the 2018 Youth Olympic Summer Games in Buenos Aires, Argentina	Br J Sports Med	Excluded	Non RCT	

PACKS-RCT	PMID	Title	Journal/Book	Excluded or Included	Exclusion reason	Randomized ("YES" if selected)
PACK1	31810972	Mental health issues and psychological factors in athletes: detection, management, effect on performance and prevention: American Medical Society for Sports Medicine Position Statement-Executive Summary	Br J Sports Med	Excluded	Non RCT	
PACK1	31834252	Effects of Exercise Training and Statin Use in People Living with Human Immunodeficiency Virus with Dyslipidemia	Med Sci Sports Exerc	Included		
PACK1	31898547	Bi-directional prospective associations between objectively measured physical activity and fundamental motor skills in children: a two-year follow-up	Int J Behav Nutr Phys Act	Excluded	Non RCT	
PACK1	31914807	Tea consumption and the risk of atherosclerotic cardiovascular disease and all-cause mortality: The China-PAR project	Eur J Prev Cardiol	Excluded	Non RCT	

PACKS-RCT	PMID	Title	Journal/Book	Excluded or Included	Exclusion reason	Randomized ("YES" if selected)
PACK1	31924625	#REDS (Relative Energy Deficiency in Sport): time for a revolution in sports culture and systems to improve athlete health and performance	Br J Sports Med	Excluded	Non RCT	
PACK1	31937125	Efficacy of extended, comprehensive outpatient cardiac rehabilitation on cardiovascular risk factors: A nationwide registry	Eur J Prev Cardiol	Excluded	Non RCT	
PACK1	31937578	Mouthguard use in youth ice hockey and the risk of concussion: nested case-control study of 315 cases	Br J Sports Med	Excluded	Non RCT	
PACK1	31937579	Diagnosis, prevention and treatment of common lower extremity muscle injuries in sport - grading the evidence: a statement paper commissioned by the Danish Society of Sports Physical Therapy (DSSF)	Br J Sports Med	Excluded	Non RCT	

PACKS-RCT	PMID	Title	Journal/Book	Excluded or Included	Exclusion reason	Randomized ("YES" if selected)
PACK1	31939911	A Randomized Community-based Exercise Training Trial in African American Men: Aerobic Plus Resistance Training and Insulin Sensitivity in African American Men	Med Sci Sports Exerc	Included		
PACK1	31939912	Feasibility and Effects on Muscle Function of an Exercise Program for Older Adults	Med Sci Sports Exerc	Included		YES
PACK1	31939915	Hypoxic Training Is Not Beneficial in Elite Athletes	Med Sci Sports Exerc	Excluded	Non RCT	
PACK1	31948454	'Jump start' childcare-based intervention to promote physical activity in pre-schoolers: six-month findings from a cluster randomised trial	Int J Behav Nutr Phys Act	Included		
PACK1	31956044	Evaluation of the bilateral function in para-athletes with spastic hemiplegia: A model-based clustering approach	J Sci Med Sport	Excluded	Non RCT	
PACK1	31959675	Rib stress injuries in the 2012-2016 (Rio) Olympiad: a cohort study of 151 Australian	Br J Sports Med	Excluded	Non RCT	

PACKS-RCT	PMID	Title	Journal/Book	Excluded or Included	Exclusion reason	Randomized ("YES" if selected)
		Rowing Team athletes for 88 773 athlete days				
PACK1	31959678	Consensus recommendations on the classification, definition and diagnostic criteria of hip-related pain in young and middle-aged active adults from the International Hip-related Pain Research Network, Zurich 2018	Br J Sports Med	Excluded	Non RCT	
PACK1	31964186	High-intensity interval training is effective and superior to moderate continuous training in patients with heart failure with preserved ejection fraction: A randomized clinical trial	Eur J Prev Cardiol	Included		
PACK1	31969347	Contact - but not foul play - dominates injury mechanisms in men's professional handball: a video match analysis of 580 injuries	Br J Sports Med	Excluded	Non RCT	
PACK1	31969348	Habitual physical activity, renal function and chronic kidney	Br J Sports Med	Excluded	Non RCT	

PACKS-RCT	PMID	Title	Journal/Book	Excluded or Included	Exclusion reason	Randomized ("YES" if selected)
		disease: a cohort study of nearly 200 000 adults				
PACK1	31975547	Effect of exercise on key pharmacokinetic parameters related to metformin absorption in healthy humans: A pilot study	Scand J Med Sci Sports	Excluded	ong intervent	
PACK1	31977108	Altered hip control during a standing knee-lift test is associated with increased risk of knee injuries	Scand J Med Sci Sports	Excluded	Non RCT	
PACK1	31980419	'My hip is damaged': a qualitative investigation of people seeking care for persistent hip pain	Br J Sports Med	Excluded	Non RCT	
PACK1	31982300	Bowling loads and injury risk in male first class county cricket: Is 'differential load' an alternative to the acute-to-chronic workload ratio?	J Sci Med Sport	Excluded	Non RCT	
PACK1	31986220	High leisure-time physical activity reduces the risk of long-term sickness absence	Scand J Med Sci Sports	Excluded	Non RCT	

PACKS-RCT	PMID	Title	Journal/Book	Excluded or Included	Exclusion reason	Randomized ("YES" if selected)
PACK1	31992545	Forty-five per cent lower acute injury incidence but no effect on overuse injury prevalence in youth floorball players (aged 12-17 years) who used an injury prevention exercise programme: two-armed parallel-group cluster randomised controlled trial	Br J Sports Med	Included		YES
PACK1	31996015	Triglyceride-containing lipoprotein sub-fractions and risk of coronary heart disease and stroke: A prospective analysis in 11,560 adults	Eur J Prev Cardiol	Excluded	Non RCT	
PACK1	31996346	Call to action: a collaborative framework to better support female rugby league players	Br J Sports Med	Excluded	Not exercise	
PACK1	31999876	Effects of progressive aquatic resistance training on symptoms and quality of life in women with knee osteoarthritis: A secondary analysis	Scand J Med Sci Sports	Included	maybe	

PACKS-RCT	PMID	Title	Journal/Book	Excluded or Included	Exclusion reason	Randomized ("YES" if selected)
PACK1	32001517	Effectiveness of an e-health tennis-specific injury prevention programme: randomised controlled trial in adult recreational tennis players	Br J Sports Med	Included		
PACK1	32005255	Socioeconomic position and the impact of increasing availability of lower energy meals vs. menu energy labelling on food choice: two randomized controlled trials in a virtual fast-food restaurant	Int J Behav Nutr Phys Act	Excluded	Not exercise	
PACK1	32008909	Injury epidemiology in Australian male professional soccer	J Sci Med Sport	Excluded	Non RCT	
PACK1	32013594	Leisure-time cross-country skiing and risk of atrial fibrillation and stroke: A prospective cohort study	Eur J Prev Cardiol	Excluded	Non RCT	
PACK1	32013597	Integrating natriuretic peptides and diastolic dysfunction to predict adverse events in high-risk	Eur J Prev Cardiol	Excluded	Non RCT	

PACKS-RCT	PMID	Title	Journal/Book	Excluded or Included	Exclusion reason	Randomized ("YES" if selected)
		asymptomatic subjects				
PACK1	32013611	Smoking status and mortality outcomes following percutaneous coronary intervention	Eur J Prev Cardiol	Excluded	Non RCT	
PACK1	32019364	LDL-cholesterol lowering with evolocumab, and outcomes according to age and sex in patients in the FOURIER Trial	Eur J Prev Cardiol	Excluded	Not exercise	
PACK1	32019370	Energy-adjusted Dietary Inflammatory Index scores predict long-term cardiovascular disease mortality and other causes of death in an ecological analysis of the Seven Countries Study	Eur J Prev Cardiol	Excluded	Non RCT	
PACK1	32024646	Fifteen-week window for recurrent muscle strains in football: a prospective cohort of 3600 muscle strains over 23 years in professional Australian rules football	Br J Sports Med	Excluded	Non RCT	

PACKS-RCT	PMID	Title	Journal/Book	Excluded or Included	Exclusion reason	Randomized ("YES" if selected)
PACK1	32034797	Skeletal maturation and growth rates are related to bone and growth plate injuries in adolescent athletics	Scand J Med Sci Sports	Excluded	Non RCT	
PACK1	32041612	The effect of moving to East Village, the former London 2012 Olympic and Paralympic Games Athletes' Village, on mode of travel (ENABLE London study, a natural experiment)	Int J Behav Nutr Phys Act	Excluded	Non RCT	
PACK1	32041634	The new child food package is associated with reduced obesity risk among formula fed infants participating in the Special Supplemental Nutrition Program for Women, Infants and Children (WIC) in Los Angeles County, California, 2003-2016	Int J Behav Nutr Phys Act	Excluded	Non RCT	
PACK1	32041640	A systematic evaluation of digital nutrition promotion websites and apps for supporting parents to	Int J Behav Nutr Phys Act	Excluded	Non RCT	

PACKS-RCT	PMID	Title	Journal/Book	Excluded or Included	Exclusion reason	Randomized ("YES" if selected)
		influence children's nutrition				
PACK1	32046955	Activity and functional readiness, not age, are the critical factors for second anterior cruciate ligament injury - the Delaware-Oslo ACL cohort study	Br J Sports Med	Excluded	Non RCT	
PACK1	32050975	Clusters of diet, physical activity, television exposure and sleep habits and their association with adiposity in preschool children: the EDEN mother-child cohort	Int J Behav Nutr Phys Act	Excluded	Non RCT	
PACK1	32050979	Reductions to main meal portion sizes reduce daily energy intake regardless of perceived normality of portion size: a 5 day cross-over laboratory experiment	Int J Behav Nutr Phys Act	Excluded	Non RCT	
PACK1	32050996	A randomized controlled trial examining consumers' perceptions and opinions on using different versions of a FoodFlip©	Int J Behav Nutr Phys Act	Excluded	Not exercise	

PACKS-RCT	PMID	Title	Journal/Book	Excluded or Included	Exclusion reason	Randomized ("YES" if selected)
		smartphone application for delivery of nutrition information				
PACK1	32059728	Patterns and predictors of exercise behavior during 24 months of follow-up after a supervised exercise program during breast cancer chemotherapy	Int J Behav Nutr Phys Act	Included		YES
PACK1	32060142	Improved reporting of overuse injuries and health problems in sport: an update of the Oslo Sport Trauma Research Center questionnaires	Br J Sports Med	Excluded	Non RCT	
PACK1	32063509	The influence of baseball pitching distance on pitching biomechanics, pitch velocity, and ball movement	J Sci Med Sport	Excluded	Non RCT	
PACK1	32079603	Consensus on a video analysis framework of descriptors and definitions by the Rugby Union Video Analysis Consensus group	Br J Sports Med	Excluded	Non RCT	

PACKS-RCT	PMID	Title	Journal/Book	Excluded or Included	Exclusion reason	Randomized ("YES" if selected)
PACK1	32081605	MRI does not effectively diagnose ulnar-sided wrist pain in elite tennis players	J Sci Med Sport	Excluded	Non RCT	
PACK1	32089004	A scoring system for predicting individual treatment effects of statins in type 2 diabetes patients on haemodialysis	Eur J Prev Cardiol	Excluded	Not exercise	
PACK1	32089005	Effectiveness of comprehensive cardiac rehabilitation in coronary artery disease patients treated according to contemporary evidence based medicine: Update of the Cardiac Rehabilitation Outcome Study (CROS-II)	Eur J Prev Cardiol	Excluded	Non RCT	
PACK1	32089006	Triglyceride concentrations and non-high-density lipoprotein cholesterol goal attainment in the ODYSSEY phase 3 trials with alirocumab	Eur J Prev Cardiol	Excluded	Not exercise	
PACK1	32089009	Comparison of Swiss and European risk algorithms for cardiovascular	Eur J Prev Cardiol	Excluded	Not exercise	

PACKS-RCT	PMID	Title	Journal/Book	Excluded or Included	Exclusion reason	Randomized ("YES" if selected)
		prevention in Switzerland				
PACK1	32089011	Association of smoking with coronary artery disease and myocardial infarction: A Mendelian randomization study	Eur J Prev Cardiol	Excluded	Not exercise	
PACK1	32102057	Cardiovascular Drift and Maximal Oxygen Uptake during Running and Cycling in the Heat	Eur J Prev Cardiol	Excluded	Wrong interventio n	
PACK1	32102059	Does Mental Fatigue Negatively Affect Outcomes of Functional Performance Tests?	Med Sci Sports Exerc	Excluded	Non RCT	
PACK1	32102060	The Prevalence of Expiratory Flow Limitation in Youth Elite Male Cyclists	Med Sci Sports Exerc	Excluded	Wrong interventio n	
PACK1	32102061	Voluntary Activation and Reflex Responses following Hamstring Strain Injury	Med Sci Sports Exerc	Excluded	Non RCT	
PACK1	32102667	Cardiorespiratory fitness and accelerometer-det ermined physical activity following one year of free-living high-intensity	Int J Behav Nutr Phys Act	Included		YES

PACKS-RCT	PMID	Title	Journal/Book	Excluded or Included	Exclusion reason	Randomized ("YES" if selected)
		interval training and moderate-intensity continuous training: a randomized trial				
PACK1	32106713	Long-term follow-up with a smartphone application improves exercise capacity post cardiac rehabilitation: A randomized controlled trial	Eur J Prev Cardiol	Excluded	Wrong interventio n	
PACK1	32114488	Why should I test my athletes in the heat several months before Tokyo 2020?	Br J Sports Med	Excluded	Non RCT	
PACK1	32118696	Chronic Exposure to Low Dose Carbon Monoxide Alters Hemoglobin Mass and VO2max	Med Sci Sports Exerc	Excluded	Not exercise	
PACK1	32127255	Exercise program and blood pressure in children: The moderating role of sedentary time	J Sci Med Sport	Included		
PACK1	32360244	Compliance with the 24-Hour movement guidelines for the early years: Cross-sectional and longitudinal associations with executive function and psychosocial	J Sci Med Sport	Excluded	Non RCT	

PACKS-RCT	PMID	Title	Journal/Book	Excluded or Included	Exclusion reason	Randomized ("YES" if selected)
		health in preschool children				
PACK2	30413429	Subclinical coronary artery disease in veteran athletes: is a new preparticipation methodology required?	Br J Sports Med	Excluded	Non RCT	
PACK2	31302602	Infographic. New Zealand rugby's community concussion initiative: keeping kiwi communities RugbySmart	Br J Sports Med	Excluded	Non RCT	
PACK2	31377723	Infographic. Risk profile for sport-related post-traumatic knee osteoarthritis	Br J Sports Med	Excluded	Non RCT	
PACK2	31377724	Infographic. Prenatal physical activity: baby steps for better health	Br J Sports Med	Excluded	Non RCT	
PACK2	31690492	Proposed injury thresholds for concussion in equestrian sports	J Sci Med Sport	Excluded	Non RCT	
PACK2	32077129	Training for success: Do simulated aerial landings replicate successful aerial landings performed in the ocean?	Scand J Med Sci Sports	Excluded	Non RCT	

PACKS-RCT	PMID	Title	Journal/Book	Excluded or Included	Exclusion reason	Randomized ("YES" if selected)
PACK2	32077144	Effects of a functional training program in patients with fibromyalgia: A 9-year prospective longitudinal cohort study	Scand J Med Sci Sports	Excluded	Non RCT	
PACK2	32096248	High-grade intramuscular tendon disruption in acute hamstring injury and return to play in Australian Football players	Scand J Med Sci Sports	Excluded	Non RCT	
PACK2	32096249	Patellofemoral alignment and geometry and early signs of osteoarthritis are associated in patellofemoral pain population	Scand J Med Sci Sports	Excluded	Non RCT	
PACK2	32100894	Concussion incidence and recovery in Swedish elite soccer - Prolonged recovery in female players	Scand J Med Sci Sports	Excluded	Non RCT	
PACK2	32122195	Increased awareness, inadequate treatment, and poor control of cardiovascular risk factors in American young adults: 2005-2015	Eur J Prev Cardiol	Excluded	Non RCT	

PACKS-RCT	PMID	Title	Journal/Book	Excluded or Included	Exclusion reason	Randomized ("YES" if selected)
PACK2	32122198	Obesity, overweight and risk for cardiovascular disease and mortality in young women	Eur J Prev Cardiol	Excluded	Non RCT	
PACK2	32122200	Underperformance of clinical risk scores in identifying vascular ultrasound-based high cardiovascular risk in systemic lupus erythematosus	Eur J Prev Cardiol	Excluded	Non RCT	
PACK2	32122203	Leisure-time cross-country skiing and the risk of venous thromboembolism: A prospective cohort study	Eur J Prev Cardiol	Excluded	Non RCT	
PACK2	32126830	Systematic Coronary Risk Evaluation estimated risk and prevalent subclinical atherosclerosis in coronary and carotid arteries: A population-based cohort analysis from the Swedish Cardiopulmonary Bioimage Study	Eur J Prev Cardiol	Excluded	Non RCT	
PACK2	32131628	Caffeinated coffee consumption and risk of atrial	Eur J Prev Cardiol	Excluded	Non RCT	

PACKS-RCT	PMID	Title	Journal/Book	Excluded or Included	Exclusion reason	Randomized ("YES" if selected)
		fibrillation in two Spanish cohorts				
PACK2	32131849	Five-year cost-effectiveness analysis of the European Fans in Training (EuroFIT) physical activity intervention for men versus no intervention	Int J Behav Nutr Phys Act	Excluded	Non RCT	
PACK2	32141109	Recording injuries only during winter competitive season underestimates injury incidence in elite alpine skiers	Scand J Med Sci Sports	Excluded	Non RCT	
PACK2	32145103	Physical fitness components in relation to attention capacity in Latin American youth with overweight and obesity	Scand J Med Sci Sports	Excluded	Non RCT	
PACK2	32151254	Prospective associations with physiological, psychosocial and educational outcomes of meeting Australian 24-Hour Movement Guidelines for the Early Years	Int J Behav Nutr Phys Act	Excluded	Non RCT	
PACK2	32153035	Lung function and oxygen saturation after participation	Scand J Med Sci Sports	Excluded	Non RCT	

PACKS-RCT	PMID	Title	Journal/Book	Excluded or Included	Exclusion reason	Randomized ("YES" if selected)
		in Norseman Xtreme Triathlon				
PACK2	32169059	Accelerometer-measured physical activity and sedentary time in a cohort of US adults followed for up to 13 years: the influence of removing early follow-up on associations with mortality	Int J Behav Nutr Phys Act	Excluded	Non RCT	
PACK2	32171647	Dietary supplementation with New Zealand blackcurrant extract enhances fat oxidation during submaximal exercise in the heat	J Sci Med Sport	Excluded	Wrong intervention	
PACK2	32173259	A comparison of acute glycaemic responses to accumulated or single bout walking exercise in apparently healthy, insufficiently active adults	J Sci Med Sport	Included		YES
PACK2	32175973	Significant Dose-Response between Exercise Adherence and Hemoglobin A1c Change	Med Sci Sports Exerc	Excluded	Non RCT	
PACK2	32175975	Neuromuscular Fatigue of Cycling Exercise in Hypoxia	Med Sci Sports Exerc	Excluded	Non RCT	

PACKS-RCT	PMID	Title	Journal/Book	Excluded or Included	Exclusion reason	Randomized ("YES" if selected)
PACK2	32176397	Education leads to a more physically active lifestyle: Evidence based on Mendelian randomization	Scand J Med Sci Sports	Excluded	Non RCT	
PACK2	32178703	Estimating physical activity from self-reported behaviours in large-scale population studies using network harmonisation: findings from UK Biobank and associations with disease outcomes	Int J Behav Nutr Phys Act	Excluded	Non RCT	
PACK2	32179511	'Theory on relativity': why we need to be 'absolute' and regulate the reporting of injury risk outcome metrics in RCTs in sport and exercise medicine (Methods Matter series)	Br J Sports Med	Excluded	Non RCT	
PACK2	32183815	Effectiveness of prescribing physical activity in parks to improve health and wellbeing - the park prescription randomized controlled trial	Int J Behav Nutr Phys Act	Included		YES

PACKS-RCT	PMID	Title	Journal/Book	Excluded or Included	Exclusion reason	Randomized ("YES" if selected)
PACK2	32195597	Primary prevention efforts are poorly developed in people at high cardiovascular risk: A report from the European Society of Cardiology EURObservational Research Programme EUROASPIRE V survey in 16 European countries	Eur J Prev Cardiol	Excluded	Non RCT	
PACK2	32195768	Casein Ingestion Does Not Increase Muscle Connective Tissue Protein Synthesis Rates	Med Sci Sports Exerc	Excluded	Non RCT	
PACK2	32195769	The Sexes Do Not Differ for Neural Responses to Submaximal Elbow Extensor Fatigue	Med Sci Sports Exerc	Excluded	Non RCT	
PACK2	32205677	Sprint and Strength Training Modulates Autophagy and Proteostasis in Aging Sprinters	Med Sci Sports Exerc	Excluded	Wrong interventio n	
PACK2	32205678	A "Step-Ramp-Step" Protocol to Identify the Maximal Metabolic Steady State	Med Sci Sports Exerc	Excluded	Non RCT	

PACKS-RCT	PMID	Title	Journal/Book	Excluded or Included	Exclusion reason	Randomized ("YES" if selected)
PACK2	32217524	Does foot mobility affect the outcome in the management of patellofemoral pain with foot orthoses versus hip exercises? A randomised clinical trial	Br J Sports Med	Included		YES
PACK2	32223322	Alcohol and heart health: The need for a randomized controlled trial	Eur J Prev Cardiol	Excluded	Non RCT	
PACK2	32228040	Profile and treatment of chronic coronary syndromes in European Society of Cardiology member countries: The ESC EORP CICD-LT registry	Eur J Prev Cardiol	Excluded	Non RCT	
PACK2	32233661	Association between ideal cardiovascular health and risk of sudden cardiac death and all-cause mortality among middle-aged men in Finland	Eur J Prev Cardiol	Excluded	Non RCT	
PACK2	32303477	Patterns of head impact exposure in men's and women's collegiate club water polo	J Sci Med Sport	Excluded	Non RCT	

PACKS-RCT	PMID	Title	Journal/Book	Excluded or Included	Exclusion reason	Randomized ("YES" if selected)
PACK2	32307227	Outcome of a neuromuscular training program on recurrent ankle sprains. Does the initial type of healthcare matter?	J Sci Med Sport	Excluded	Wrong interventio n	
PACK2	32345543	Effect of a novel low volume, high intensity concurrent training regimen on recruit fitness and resilience	J Sci Med Sport	Excluded	Non RCT	
PACK3	30842104	Association between physical activity and sedentary behaviour on carotid atherosclerotic plaques: an epidemiological and histological study in 90 asymptomatic patients	Br J Sports Med	Excluded	Non RCT	
PACK3	31362925	Eighty-two per cent of male professional football (soccer) players return to play at the previous level two seasons after Achilles tendon rupture treated with surgical repair	Br J Sports Med	Excluded	Non RCT	

PACKS-RCT	PMID	Title	Journal/Book	Excluded or Included	Exclusion reason	Randomized ("YES" if selected)
PACK3	31492676	Does disallowing body checking in non-elite 13- to 14-year-old ice hockey leagues reduce rates of injury and concussion? A cohort study in two Canadian provinces	Br J Sports Med	Excluded	Non RCT	
PACK3	31562122	Towards better evidence-informed global action: lessons learnt from the Lancet series and recent developments in physical activity and public health	Br J Sports Med	Excluded	Non RCT	
PACK3	31649020	Infographic. Developing home-based cardiac rehabilitation for people post-transient ischaemic attack (TIA) and ischaemic stroke	Br J Sports Med	Excluded	Non RCT	
PACK3	31810973	Sports concussions: can head impact sensors help biomedical engineers to design better headgear?	Br J Sports Med	Excluded	Non RCT	
PACK3	32113577	Performance in the heat: Health complaints and	J Sci Med Sport	Excluded	Non RCT	

PACKS-RCT	PMID	Title	Journal/Book	Excluded or Included	Exclusion reason	Randomized ("YES" if selected)
		heat stress prevention strategies as predictors				
PACK3	32150772	Inter-individual variation in response to resistance training in cardiometabolic health indicators	Scand J Med Sci Sports	Included		YES
PACK3	32187403	Carbohydrates do not accelerate force recovery after glycogen-depleting followed by high-intensity exercise in humans	Scand J Med Sci Sports	Excluded	Non RCT	
PACK3	32187716	Dynamic knee valgus in competitive alpine skiers: Observation from youth to elite and influence of biological maturation	Scand J Med Sci Sports	Excluded	Non RCT	
PACK3	32201975	Developing web-based health guidance for coaches and parents in child athletics (track and field)	Scand J Med Sci Sports	Excluded	Non RCT	
PACK3	32201993	The relationship between cam morphology and hip and groin symptoms and signs in young male football players	Scand J Med Sci Sports	Excluded	Non RCT	

PACKS-RCT	PMID	Title	Journal/Book	Excluded or Included	Exclusion reason	Randomized ("YES" if selected)
PACK3	32241819	Lower nationwide rates of arthroscopic procedures in 2016 compared with 1997 (634925 total arthroscopic procedures): has the tide turned?	Br J Sports Med	Excluded	Non RCT	
PACK3	32243644	Acute effects of breaking up prolonged sedentary time on cardiovascular disease risk markers in adults with paraplegia	Scand J Med Sci Sports	Excluded	Non RCT	
PACK3	32246553	Sports participation and sports injuries in Dutch boys with haemophilia	Scand J Med Sci Sports	Excluded	Non RCT	
PACK3	32250171	The Moderate Alcohol and Cardiovascular Health Trial (MACH15): Design and methods for a randomized trial of moderate alcohol consumption and cardiometabolic risk	Eur J Prev Cardiol	Excluded	Not exercise	
PACK3	32267032	Systemic and muscular responses to effort-matched short intervals and long intervals in elite cyclists	Scand J Med Sci Sports	Excluded	Non RCT	

PACKS-RCT	PMID	Title	Journal/Book	Excluded or Included	Exclusion reason	Randomized ("YES" if selected)
PACK3	32268801	The Coronary ARteriogenesis with combined Heparin and EXercise therapy in chronic refractory Angina (CARHEXA) trial: A double-blind, randomized, placebo-controlled stress echocardiographic study	Eur J Prev Cardiol	Included		YES
PACK3	32270698	Physical inactivity and cardiovascular disease at the time of coronavirus disease 2019 (COVID-19)	Eur J Prev Cardiol	Excluded	Non RCT	
PACK3	32276571	Non-genetic risk factors for atrial fibrillation are equally important in both young and old age: A nationwide population-based study	Eur J Prev Cardiol	Excluded	Non RCT	
PACK3	32276576	Physical activity attenuates cardiovascular risk and mortality in men and women with and without the metabolic syndrome - a 20-year follow-up of a population-based	Eur J Prev Cardiol	Excluded	Non RCT	

PACKS-RCT	PMID	Title	Journal/Book	Excluded or Included	Exclusion reason	Randomized ("YES" if selected)
		cohort of 60-year-olds				
PACK3	32276577	Discordant responses of plasma low-density lipoprotein cholesterol and lipoprotein(a) to alirocumab: A pooled analysis from 10 ODYSSEY Phase 3 studies	Eur J Prev Cardiol	Excluded	Non RCT	
PACK3	32276925	Poor functional performance 1 year after ACL reconstruction increases the risk of early osteoarthritis progression	Br J Sports Med	Excluded	Non RCT	
PACK3	32283957	HDL-C is associated with mortality from all causes, cardiovascular disease and cancer in a J-shaped dose-response fashion: a pooled analysis of 37 prospective cohort studies	Eur J Prev Cardiol	Excluded	Non RCT	
PACK3	32295621	Like me, like you - relative importance of peers and siblings on children's fast food consumption and screen time but not sports club participation depends on age	Int J Behav Nutr Phys Act	Excluded	Non RCT	

PACKS-RCT	PMID	Title	Journal/Book	Excluded or Included	Exclusion reason	Randomized ("YES" if selected)
PACK3	32297361	Bidirectional associations between fitness and fatness in youth: A longitudinal study	Scand J Med Sci Sports	Excluded	Non RCT	
PACK3	32301349	Cost-effectiveness of rivaroxaban plus aspirin (dual pathway inhibition) for prevention of ischaemic events in patients with cardiovascular disease: on top optimisation of secondary prevention medication in the context of COVID-19 pandemia	Eur J Prev Cardiol	Excluded	Non RCT	
PACK3	32303522	Athletes as community; athletes in community: covid-19, sporting mega-events and athlete health protection	Br J Sports Med	Excluded	Non RCT	
PACK3	32306757	Subclinical progression of systemic sclerosis-related cardiomyopathy	Eur J Prev Cardiol	Excluded	Non RCT	
PACK3	32321285	Improvements in exercise capacity of older adults during cardiac rehabilitation	Eur J Prev Cardiol	Excluded	Non RCT	

PACKS-RCT	PMID	Title	Journal/Book	Excluded or Included	Exclusion reason	Randomized ("YES" if selected)
PACK3	32336568	No differences in tandem gait performance between male and female athletes acutely post-concussion	J Sci Med Sport	Excluded	Non RCT	
PACK3	32340462	Greater burden of risk factors and less effect of cardiac rehabilitation in elderly with low educational attainment: The Eu-CaRE study	Eur J Prev Cardiol	Excluded	Non RCT	
PACK3	32341022	Preventing injuries in alpine skiing giant slalom by shortening the vertical distance between the gates rather than increasing the horizontal gate offset to control speed	Br J Sports Med	Excluded	Non RCT	
PACK3	32349761	Stand Out in Class: restructuring the classroom environment to reduce sitting time - findings from a pilot cluster randomised controlled trial	Int J Behav Nutr Phys Act	Excluded	Not exercise	
PACK3	32349922	Kinematic and kinetic comparison between American and Japanese collegiate pitchers	J Sci Med Sport	Excluded	Non RCT	

PACKS-RCT	PMID	Title	Journal/Book	Excluded or Included	Exclusion reason	Randomized ("YES" if selected)
PACK3	32359939	Incidence, prevalence and consequences of illness in academy rugby league players	J Sci Med Sport	Excluded	Non RCT	
PACK3	32359940	The effect of physical fatigue on tackling technique in Rugby Union	J Sci Med Sport	Excluded	Non RCT	
PACK3	32362482	Can the Y balance test identify those at risk of contact or non-contact lower extremity injury in adolescent and collegiate Gaelic games?	J Sci Med Sport	Excluded	Non RCT	
PACK3	32371120	Impact of low-volume concurrent strength training distribution on muscular adaptation	J Sci Med Sport	Included		YES
PACK3	32451269	Obstructive sleep apnea in professional rugby league athletes: An exploratory study	J Sci Med Sport	Excluded	Non RCT	
PACK4	30396293	Association between physical activity, occupational sitting time and mortality in a general population: An 18-year prospective survey	Eur J Prev Cardiol	Excluded	Non RCT	

PACKS-RCT	PMID	Title	Journal/Book	Excluded or Included	Exclusion reason	Randomized ("YES" if selected)
		in Tanushimaru, Japan				
PACK4	31434660	How sport and exercise medicine research can protect athlete health and promote athlete performance	Br J Sports Med	Excluded	Non RCT	
PACK4	31630091	A novel hamstring strain injury prevention system: post-match strength testing for secondary prevention in football	Br J Sports Med	Excluded	Non RCT	
PACK4	31924536	The plateau at V˙O(2max) is associated with anaerobic alleles	J Sci Med Sport	Excluded	Non RCT	
PACK4	31925768	Intra-articular Injection Administration in UK Ex-professional Footballers During Their Playing Careers and the Association with Post-career Knee Osteoarthritis	Sports Med	Excluded	Non RCT	
PACK4	31928881	Determinants of hamstring fascicle length in professional rugby league athletes	J Sci Med Sport	Excluded	Non RCT	
PACK4	31985574	Dual-Task Gait Recovery after Concussion among Female and Male Collegiate Athletes	Med Sci Sports Exerc	Excluded	Non RCT	

PACKS-RCT	PMID	Title	Journal/Book	Excluded or Included	Exclusion reason	Randomized ("YES" if selected)
PACK4	32077066	Recommendations on the Appropriate Level of Medical Support at Ultramarathons	Sports Med	Excluded	Non RCT	
PACK4	32278323	The effect of previous shoulder pain on supraspinatus tendon thickness changes following swimming practice	Scand J Med Sci Sports	Excluded	Non RCT	
PACK4	32279363	Effects of the FIFA 11+ on injury prevention in amateur futsal players	Scand J Med Sci Sports	Included		YES
PACK4	32293738	Does an effective shoulder injury prevention program affect risk factors in handball? A randomized controlled study	Scand J Med Sci Sports	Included		YES
PACK4	32302455	Injuries and illnesses in Swedish Paralympic athletes-A 52-week prospective study of incidence and risk factors	Scand J Med Sci Sports	Excluded	Non RCT	
PACK4	32311152	Characterizing the resting metabolic rate ratio in ovulatory exercising women over 12 months	Scand J Med Sci Sports	Excluded	Non RCT	

PACKS-RCT	PMID	Title	Journal/Book	Excluded or Included	Exclusion reason	Randomized ("YES" if selected)
PACK4	32311175	Athletes with a concussion history in the last two years have impairments in dynamic balance performance	Scand J Med Sci Sports	Excluded	Non RCT	
PACK4	32343021	Anterior cruciate ligament reconstructed individuals demonstrate slower reactions during a dynamic postural task	Scand J Med Sci Sports	Excluded	Non RCT	
PACK4	32350931	Effect of a tailored home-based exercise program in patients with systemic sclerosis: A randomized controlled trial	Scand J Med Sci Sports	Included		
PACK4	32353906	The "11 for Health in Denmark" intervention in 10- to 12-year-old Danish girls and boys and its effects on well-being-A large-scale cluster RCT	Scand J Med Sci Sports	Included		YES
PACK4	32366796	Acute Photobiomodulation by LED Does Not Alter Muscle Fatigue and Cycling Performance	Med Sci Sports Exerc	Excluded	Non RCT	

PACKS-RCT	PMID	Title	Journal/Book	Excluded or Included	Exclusion reason	Randomized ("YES" if selected)
PACK4	32366799	Power of Words: Influence of Preexercise Information on Hypoalgesia after Exercise-Randomized Controlled Trial	Med Sci Sports Exerc	Excluded	Wrong intervention	
PACK4	32371524	Statement on methods in sport injury research from the 1st METHODS MATTER Meeting, Copenhagen, 2019	Br J Sports Med	Excluded	Non RCT	
PACK4	32375550	Prevalence and determinants of the precursor stages of heart failure: results from the population-based STAAB cohort study	Eur J Prev Cardiol	Excluded	Non RCT	
PACK4	32376674	Prevalence of therapeutic use exemptions at the Olympic Games and association with medals: an analysis of data from 2010 to 2018	Br J Sports Med	Excluded	Non RCT	
PACK4	32380860	Extracellular vesicle species differentially affect endothelial cell functions and differentially respond to exercise training in patients with	Eur J Prev Cardiol	Excluded	Non RCT	

PACKS-RCT	PMID	Title	Journal/Book	Excluded or Included	Exclusion reason	Randomized ("YES" if selected)
		chronic coronary syndromes				
PACK4	32389024	Preexisting coronary heart disease and susceptibility to long-term effects of traffic-related air pollution: A matched cohort analysis	Eur J Prev Cardiol	Excluded	Non RCT	
PACK4	32391719	Cardiovascular disease and COVID-19: les liaisons dangereuses	Eur J Prev Cardiol	Excluded	Non RCT	
PACK4	32393304	Longitudinal association between body mass index and physical activity among adolescents with different parental risk: a parallel latent growth curve modeling approach	Int J Behav Nutr Phys Act	Excluded	Non RCT	
PACK4	32393359	Mother-child dyadic influences of affect on everyday movement behaviors: evidence from an ecological momentary assessment study	Int J Behav Nutr Phys Act	Excluded	Non RCT	

PACKS-RCT	PMID	Title	Journal/Book	Excluded or Included	Exclusion reason	Randomized ("YES" if selected)
PACK4	32398158	Soft drink intake is associated with weight gain, regardless of physical activity levels: the health workers cohort study	Int J Behav Nutr Phys Act	Excluded	Non RCT	
PACK4	32403173	Increased oxygen extraction and mitochondrial protein expression after small muscle mass endurance training	Scand J Med Sci Sports	Excluded	Non RCT	
PACK4	32404175	Association between time-weighted activity space-based exposures to fast food outlets and fast food consumption among young adults in urban Canada	Int J Behav Nutr Phys Act	Excluded	Non RCT	
PACK4	32408762	Rationale, design and baseline characteristics of the MyoVasc study: A prospective cohort study investigating development and progression of heart failure	Eur J Prev Cardiol	Excluded	Non RCT	

PACKS-RCT	PMID	Title	Journal/Book	Excluded or Included	Exclusion reason	Randomized ("YES" if selected)
PACK4	32414381	Results of caring and reaching for health (CARE): a cluster-randomized controlled trial assessing a worksite wellness intervention for child care staff	Int J Behav Nutr Phys Act	Included		YES
PACK4	32416971	In the frame, road map for Australian sport on an uncertain journey through COVID-19	J Sci Med Sport	Excluded	Non RCT	
PACK4	32418489	Cardiac patient care during a pandemic: how to reorganise a heart failure unit at the time of COVID-19	Eur J Prev Cardiol	Excluded	Non RCT	
PACK4	32418804	A deep dive into testing and management of COVID-19 for Australian high performance and professional sport	J Sci Med Sport	Excluded	Non RCT	
PACK4	32451268	The Australian Institute of Sport framework for rebooting sport in a COVID-19 environment	J Sci Med Sport	Excluded	Non RCT	
PACK4	32456979	A 4-week endurance training program improves tolerance to mental exertion in untrained individuals	J Sci Med Sport	Excluded	Non RCT	

PACKS-RCT	PMID	Title	Journal/Book	Excluded or Included	Exclusion reason	Randomized ("YES" if selected)
PACK4	32461050	Hamstring strength and architectural adaptations following inertial flywheel resistance training	J Sci Med Sport	Excluded		YES
PACK4	32467150	Specifying the treatment targets of exercise interventions: do we?	Br J Sports Med	Excluded	Non RCT	
PACK4	32471784	Variations in the TRPV1 gene are associated to exertional heat stroke	J Sci Med Sport	Excluded	Non RCT	
PACK4	32471786	Proprioceptive abilities improve in expert skiers and snowboarders from early-season to mid-season	J Sci Med Sport	Excluded	Non RCT	
PACK4	32472928	Effect of an Innovative Mattress and Cryotherapy on Sleep after an Elite Rugby Match	Med Sci Sports Exerc	Excluded	Non RCT	
PACK4	32472930	Left Ventricular Fibrosis in Middle-Age Athletes and Physically Active Adults	Med Sci Sports Exerc	Excluded	Non RCT	
PACK4	32475780	Physical literacy profiles are associated with differences in children's physical activity participation: A	J Sci Med Sport	Excluded	Non RCT	

PACKS-RCT	PMID	Title	Journal/Book	Excluded or Included	Exclusion reason	Randomized ("YES" if selected)
		latent profile analysis approach				
PACK4	32482610	Using machine learning to improve our understanding of injury risk and prediction in elite male youth football players	J Sci Med Sport	Excluded	Non RCT	
PACK4	32482611	Aluminium salt-based antiperspirant coated prosthesis liners do not suppress local sweating during moderate intensity exercise in hot and temperate conditions	J Sci Med Sport	Excluded	Not exercise	
PACK4	32507623	Aviation Rescue Firefighters physical fitness and predictors of task performance	J Sci Med Sport	Excluded	Non RCT	
PACK4	32507624	Beetroot juice supplementation increases concentric and eccentric muscle power output. Original investigation	J Sci Med Sport	Excluded	Not exercise	
PACK4	32540320	Ski boot canting adjustments affect kinematic, kinetic, and postural control measures associated with fall and injury risk	J Sci Med Sport	Excluded	Not exercise	

PACKS-RCT	PMID	Title	Journal/Book	Excluded or Included	Exclusion reason	Randomized ("YES" if selected)
PACK4	32546436	The NICA injury surveillance system: Design, methodology and preliminary data of a prospective, longitudinal study of injuries in youth cross country mountain bike racing	J Sci Med Sport	Excluded	Non RCT	
PACK5	31406018	Collider bias (aka sample selection bias) in observational studies: why the effects of hyperandrogenism in elite women's sport are likely underestimated	Br J Sports Med	Excluded	Non RCT	
PACK5	31857337	Economic evaluations in 'non-inferiority' trials: can costs guide decisions between surgical and non-surgical interventions?	Br J Sports Med	Excluded	Non RCT	
PACK5	31892534	Back to basics: 10 facts every person should know about back pain	Br J Sports Med	Excluded	Non RCT	
PACK5	31919035	Predictive modelling of the physical demands during training and competition in professional soccer players	J Sci Med Sport	Excluded	Non RCT	

PACKS-RCT	PMID	Title	Journal/Book	Excluded or Included	Exclusion reason	Randomized ("YES" if selected)
PACK5	31928883	Are nutritional supplements a gateway to doping use in competitive team sports? The roles of achievement goals and motivational regulations	J Sci Med Sport	Excluded	Non RCT	
PACK5	31970718	Effect of a Concussion on Anterior Cruciate Ligament Injury Risk in a General Population	Sports Med	Excluded	Non RCT	
PACK5	31972629	No Clinical Predictors of Postconcussion Musculoskeletal Injury in College Athletes	Med Sci Sports Exerc	Excluded	1	
PACK5	31972630	Effect of High-Intensity Training and Asthma on the V˙O2 Kinetics of Adolescents	Med Sci Sports Exerc	Included		
PACK5	31977640	Pectin-Alginate Does Not Further Enhance Exogenous Carbohydrate Oxidation in Running	Med Sci Sports Exerc	Excluded	Not exercise	
PACK5	32028455	Footwear and Cadence Affect Gait Variability in Runners with Patellofemoral Pain	Med Sci Sports Exerc	Excluded	Not exercise	

PACKS-RCT	PMID	Title	Journal/Book	Excluded or Included	Exclusion reason	Randomized ("YES" if selected)
PACK5	32407583	Proprioceptive neuromuscular facilitation improves pain and descending mechanics among elderly with knee osteoarthritis	Scand J Med Sci Sports	Included		YES
PACK5	32416625	Muscle contributions to tibiofemoral shear forces and valgus and rotational joint moments during single leg drop landing	Scand J Med Sci Sports	Excluded	Non RCT	
PACK5	32421886	Training for a (half-)marathon: Training volume and longest endurance run related to performance and running injuries	Scand J Med Sci Sports	Excluded	Non RCT	
PACK5	32426888	Preschoolers exhibit greater on-task behavior following physically active lessons on the approximate number system	Scand J Med Sci Sports	Excluded	Non RCT	
PACK5	32427398	Step by step: Association of device-measured daily steps with all-cause mortality-A prospective cohort Study	Scand J Med Sci Sports	Excluded	Non RCT	

PACKS-RCT	PMID	Title	Journal/Book	Excluded or Included	Exclusion reason	Randomized ("YES" if selected)
PACK5	32427750	Association between Objective Activity Intensity and Heart Rate Variability: Cardiovascular Disease Risk Factor Mediation (CARDIA)	Med Sci Sports Exerc	Excluded	Non RCT	
PACK5	32432844	Difference in Interoception between Long-Distance Runners and Sprinters: An Event-related Potential Study	Med Sci Sports Exerc	Excluded	Non RCT	
PACK5	32474974	Comparison of balance performance between masters Olympic weightlifters and runners	Scand J Med Sci Sports	Excluded	Non RCT	
PACK5	32474979	Isometric exercises do not provide immediate pain relief in Achilles tendinopathy: A quasi-randomized clinical trial	Scand J Med Sci Sports	Excluded	Non RCT	
PACK5	32475157	Exercise in the Severe Acute Respiratory Syndrome Coronavirus-2 (SARS-CoV-2) era: A Question and Answer session with the experts Endorsed by the	Eur J Prev Cardiol	Excluded	Non RCT	

PACKS-RCT	PMID	Title	Journal/Book	Excluded or Included	Exclusion reason	Randomized ("YES" if selected)
		section of Sports Cardiology & Exercise of the European Association of Preventive Cardiology (EAPC)				
PACK5	32479128	Age matters: differences in exercise-induced cardiovascular remodelling in young and middle aged healthy sedentary individuals	Eur J Prev Cardiol	Excluded	Non RCT	
PACK5	32487121	Effects of snack portion size on anticipated and experienced hunger, eating enjoyment, and perceived healthiness among children	Int J Behav Nutr Phys Act	Excluded	Non RCT	
PACK5	32488898	Motives and barriers to initiation and sustained exercise adherence in a fitness club setting-A one-year follow-up study	Scand J Med Sci Sports	Excluded	Non RCT	
PACK5	32492220	The epidemiology of kicking injuries in professional Rugby Union: A 15-season prospective study	Scand J Med Sci Sports	Excluded	Non RCT	

PACKS-RCT	PMID	Title	Journal/Book	Excluded or Included	Exclusion reason	Randomized ("YES" if selected)
PACK5	32492229	The structural validity of the IKDC and its relationship with quality of life following ACL reconstruction	Scand J Med Sci Sports	Excluded	Non RCT	
PACK5	32501613	The causal pathway effects of a physical activity intervention on adiposity in children: The KISS Study cluster randomized clinical trial	Scand J Med Sci Sports	Included		YES
PACK5	32502306	Motivational processes during physical endurance tasks	Scand J Med Sci Sports	Excluded	Non RCT	
PACK5	32502323	Health problems in youth competitive alpine skiing: A 12-month observation of 155 athletes around the growth spurt	Scand J Med Sci Sports	Excluded	Non RCT	
PACK5	32520868	Associations of Exercise Types with All-Cause Mortality among U.S. Adults	Med Sci Sports Exerc	Excluded	Non RCT	
PACK5	32520869	Nonsteroidal Anti-inflammatory Drugs and Cardiovascular Risk in American Football	Med Sci Sports Exerc	Excluded	Non RCT	
PACK5	32520871	Resistance Training during Chemotherapy with Doxorubicin	Med Sci Sports Exerc	Excluded	Animals	

PACKS-RCT	PMID	Title	Journal/Book	Excluded or Included	Exclusion reason	Randomized ("YES" if selected)
PACK5	32520873	Metabolic Flexibility during Exercise in Children with Obesity and Matched Controls	Med Sci Sports Exerc	Excluded	Non RCT	
PACK5	32520874	Effects of the Turbine™ on Ventilatory and Sensory Responses to Incremental Cycling	Med Sci Sports Exerc	Excluded	Not exercise	
PACK5	32520876	Exercise and Protein Effects on Strength and Function with Weight Loss in Older Women	Med Sci Sports Exerc	Included		YES
PACK5	32532845	Clinical outcomes in adult athletes with hypertrophic cardiomyopathy: a 7-year follow-up study	Br J Sports Med	Excluded	Non RCT	
PACK5	32538495	Caffeine improves various aspects of athletic performance in adolescents independent of their 163 C > A CYP1A2 genotypes	Scand J Med Sci Sports	Excluded	Non RCT	
PACK5	32555019	Vascular Protection by Exercise in Obesity: Inflammasome-associated Mechanisms	Med Sci Sports Exerc	Excluded	Animals	
PACK5	32555020	Development of Concise Physical Performance Test	Med Sci Sports Exerc	Excluded	Non RCT	

PACKS-RCT	PMID	Title	Journal/Book	Excluded or Included	Exclusion reason	Randomized ("YES" if selected)
		Batteries in Young Athletes				
PACK5	32555024	Metabolic Impact of Breaking Up Prolonged Sitting with Stair Climbing Exercise Snacks	Med Sci Sports Exerc	Excluded	Wrong interventio n	
PACK5	32555025	An Intervention for Mental Health Literacy and Resilience in Organized Sports	Med Sci Sports Exerc	Excluded	Not exercise	
PACK5	32555026	High-Intensity Shoulder Abduction Exercise in Subacromial Pain Syndrome	Med Sci Sports Exerc	Excluded	ong intervent	
PACK5	32555027	Effects of Land- vs Water-Walking Interventions on Vascular Function in Older Adults	Med Sci Sports Exerc	Included		YES
PACK5	32571334	A tale of two cities: the cost, price-differential and affordability of current and healthy diets in Sydney and Canberra, Australia	Int J Behav Nutr Phys Act	Excluded	Non RCT	
PACK5	32586943	Epidemiology of injury and illness in 153 Australian international-level rowers over eight international seasons	Br J Sports Med	Excluded	Non RCT	
PACK5	32594762	Added sugar intake is associated with pericardial adipose tissue volume	Eur J Prev Cardiol	Excluded	Non RCT	

PACKS-RCT	PMID	Title	Journal/Book	Excluded or Included	Exclusion reason	Randomized ("YES" if selected)
PACK5	32597206	Recommendations for participation in leisure-time physical activity and competitive sports in patients with arrhythmias and potentially arrhythmogenic conditions: Part 1: Supraventricular arrhythmias. A position statement of the Section of Sports Cardiology and Exercise from the European Association of Preventive Cardiology (EAPC) and the European Heart Rhythm Association (EHRA), both associations of the European Society of Cardiology	Eur J Prev Cardiol	Excluded	Non RCT	
PACK5	32599670	Muscle contractile properties of cancer patients receiving chemotherapy: Assessment of feasibility and exercise effects	Scand J Med Sci Sports	Included		YES

PACKS-RCT	PMID	Title	Journal/Book	Excluded or Included	Exclusion reason	Randomized ("YES" if selected)
PACK5	32620352	Is the gut microbiota bacterial abundance and composition associated with intestinal epithelial injury, systemic inflammatory profile, and gastrointestinal symptoms in response to exertional-heat stress?	J Sci Med Sport	Excluded	Non RCT	
PACK5	32624441	Changes in exertion-related symptoms in adults and youth who have sustained a sport-related concussion	J Sci Med Sport	Excluded	Non RCT	
PACK5	32624442	Nutrient intake, meal timing and sleep in elite male Australian football players	J Sci Med Sport	Excluded	Non RCT	
PACK5	32631774	Knee injury prevention in alpine skiing. A technological paradigm shift towards a mechatronic ski binding	J Sci Med Sport	Excluded	Non RCT	
PACK5	32646746	Physiological factors determining downhill vs uphill	J Sci Med Sport	Excluded	Wrong interventio n	

PACKS-RCT	PMID	Title	Journal/Book	Excluded or Included	Exclusion reason	Randomized ("YES" if selected)
		running endurance performance				
PACK6	31399427	Letting the cat out of the bag: athletes, coaches and physiotherapists share their perspectives on injury prevention in elite sports	Br J Sports Med	Excluded	Non RCT	
PACK6	31871015	Identifying the 'incredible'! Part 1: assessing the risk of bias in outcomes included in systematic reviews	Br J Sports Med	Excluded	Non RCT	
PACK6	31871016	Elephant in the room: how much pain is ok? If physiotherapy exercise RCTs do not report it, we will never answer the question	Br J Sports Med	Excluded	Non RCT	
PACK6	31977632	Nonexercise Equations for Determining Change in Cardiorespiratory Fitness	Med Sci Sports Exerc	Included		YES
PACK6	31977634	Timing of Vaccination after Training: Immune Response and Side Effects in Athletes	Med Sci Sports Exerc	Excluded	Non RCT	
PACK6	31977638	Exercise Improves Video Game Performance: A Win-Win Situation	Med Sci Sports Exerc	Excluded	Non RCT	

PACKS-RCT	PMID	Title	Journal/Book	Excluded or Included	Exclusion reason	Randomized ("YES" if selected)
PACK6	31977642	High-Intensity Training and Saffron: Effects on Breast Cancer-related Gene Expression	Med Sci Sports Exerc	Excluded	Non RCT	
PACK6	31977643	Neck and Inspiratory Muscle Recruitment during Inspiratory Loading and Neck Flexion	Med Sci Sports Exerc	Excluded	Non RCT	
PACK6	31985575	Modification of Neuromuscular Junction Protein Expression by Exercise and Doxorubicin	Med Sci Sports Exerc	Excluded	Non RCT	
PACK6	32125668	Effects of Autograft Types on Muscle Strength and Functional Capacity in Patients Having Anterior Cruciate Ligament Reconstruction: A Randomized Controlled Trial	Sports Med	Excluded	Not exercise	
PACK6	32168104	Increased Anandamide and Decreased Pain and Depression after Exercise in Fibromyalgia	Med Sci Sports Exerc	Excluded	Non RCT	
PACK6	32306240	Is Open-Label Placebo a New Ergogenic Aid? A Commentary on Existing Studies	Sports Med	Excluded	Non RCT	

PACKS-RCT	PMID	Title	Journal/Book	Excluded or Included	Exclusion reason	Randomized ("YES" if selected)
		and Guidelines for Future Research				
PACK6	32350816	Correction to: Is Open-Label Placebo a New Ergogenic Aid? A Commentary on Existing Studies and Guidelines for Future Research	Sports Med	Excluded	Non RCT	
PACK6	32583735	Editor's presentation: 'Les liaisones dangerouses. The heart in the time of COVID-19'	Eur J Prev Cardiol	Excluded	Non RCT	
PACK6	32608527	Simplified indices of exercise tolerance in patients with multiple sclerosis and healthy subjects: A case-control study	Scand J Med Sci Sports	Excluded	Non RCT	
PACK6	32611251	The effects of transitions in metabolic health and obesity status on incident cardiovascular disease: Insights from a general Chinese population	Eur J Prev Cardiol	Excluded	Non RCT	
PACK6	32615645	How many runners with new-onset Achilles tendinopathy develop persisting symptoms? A large prospective cohort study	Scand J Med Sci Sports	Excluded	Non RCT	

PACKS-RCT	PMID	Title	Journal/Book	Excluded or Included	Exclusion reason	Randomized ("YES" if selected)
PACK6	32623903	Predictive ability of longitudinal changes in PRECISE-DAPT score in patients on dual antiplatelet therapy: The RE-SCORE multicentre prospective registry	Eur J Prev Cardiol	Excluded	Non RCT	
PACK6	32631366	Prompting consumers to make healthier food choices in hospitals: a cluster randomised controlled trial	Int J Behav Nutr Phys Act	Excluded	Not exercise	
PACK6	32635766	Antihypertensive strategies and hypertension control in Sub-Saharan Africa	Eur J Prev Cardiol	Excluded	Non RCT	
PACK6	32645227	Effectiveness of blood flow-restricted slow walking on mobility in severe multiple sclerosis: A pilot randomized trial	Scand J Med Sci Sports	Included		YES
PACK6	32646302	Beyond randomised studies: recommendations for cardiac rehabilitation following repair of thoracic aortic	Eur J Prev Cardiol	Excluded	Non RCT	

PACKS-RCT	PMID	Title	Journal/Book	Excluded or Included	Exclusion reason	Randomized ("YES" if selected)
		aneurysm or dissection				
PACK6	32646435	Diurnal patterns of sedentary behavior and changes in physical function over time among older women: a prospective cohort study	Int J Behav Nutr Phys Act	Excluded	Non RCT	
PACK6	32650787	Physical activity-mediated associations between perceived neighborhood social environment and depressive symptoms among Jackson Heart Study participants	Int J Behav Nutr Phys Act	Excluded	Non RCT	
PACK6	32653248	Modifying bowling kinematics in cricket pace bowlers with exercise-based injury prevention: A cluster-randomised controlled trial	J Sci Med Sport	Excluded	Not exercise	
PACK6	32653249	Relationship between the 24-Hour Movement Guidelines and fundamental motor skills in preschoolers	J Sci Med Sport	Excluded	Non RCT	
PACK6	32660833	The effect of astaxanthin supplementation on performance	J Sci Med Sport	Excluded	Not exercise	

PACKS-RCT	PMID	Title	Journal/Book	Excluded or Included	Exclusion reason	Randomized ("YES" if selected)
		and fat oxidation during a 40 km cycling time trial				
PACK6	32680841	A Swedish primary healthcare prevention programme focusing on promotion of physical activity and a healthy lifestyle reduced cardiovascular events and mortality: 22-year follow-up of 5761 study participants and a reference group	Br J Sports Med	Excluded	Non RCT	
PACK6	32681596	Caffeine increases strength and power performance in resistance-trained females during early follicular phase	Scand J Med Sci Sports	Excluded	Not exercise	
PACK6	32681665	Effects of velocity loss in the bench press exercise on strength gains, neuromuscular adaptations and muscle hypertrophy	Scand J Med Sci Sports	Included		YES
PACK6	32689834	Eligibility for PCSK9 inhibitors based on the 2019 ESC/EAS and 2018 ACC/AHA guidelines	Eur J Prev Cardiol	Excluded	Non RCT	

PACKS-RCT	PMID	Title	Journal/Book	Excluded or Included	Exclusion reason	Randomized ("YES" if selected)
PACK6	32690043	The physical activity paradox revisited: a prospective study on compositional accelerometer data and long-term sickness absence	Int J Behav Nutr Phys Act	Excluded	Non RCT	
PACK6	32694363	Cardiorespiratory Fitness, Serum Vitamin D, and Prevalence of Metabolic Syndrome in Men	Med Sci Sports Exerc	Excluded	Non RCT	
PACK6	32694365	Biomechanical Effects of Blood Flow Restriction Training after ACL Reconstruction	Med Sci Sports Exerc	Excluded	Non RCT	
PACK6	32694366	Effect of Functional Impact Training on Body Composition, Bone Mineral Density, and Strength in Breast Cancer Survivors	Med Sci Sports Exerc	Included		YES
PACK6	32694367	An Untargeted Urine Metabolomics Approach for Autologous Blood Transfusion Detection	Med Sci Sports Exerc	Excluded	Not exercise	
PACK6	32694369	Altered Position Sense after Submaximal Eccentric Exercise-inducing Central Fatigue	Med Sci Sports Exerc	Excluded	Non RCT	
PACK6	32694371	Prognostic Value of Leg Muscle	Med Sci Sports Exerc	Excluded	Non RCT	

PACKS-RCT	PMID	Title	Journal/Book	Excluded or Included	Exclusion reason	Randomized ("YES" if selected)
		Strength in Acute Heart Failure Syndrome				
PACK6	32694373	Accelerated Muscle Recovery in Baseball Pitchers Using Phase Change Material Cooling	Med Sci Sports Exerc	Excluded	Not exercise	
PACK6	32694374	Muscle Glycogen Utilization during Exercise following Ingestion of Alcohol	Med Sci Sports Exerc	Excluded	Non RCT	
PACK6	32694376	Ventilatory Responsiveness during Exercise and Performance Impairment in Acute Hypoxia	Med Sci Sports Exerc	Excluded	Non RCT	
PACK6	32701872	Force and Rate Metrics Provide Return-to-Sport Criterion after ACL Reconstruction	Med Sci Sports Exerc	Excluded	Non RCT	
PACK6	32701873	Kinematic Sequence Classification and the Relationship to Pitching Limb Torques	Med Sci Sports Exerc	Excluded	Non RCT	
PACK6	32701874	Supplementary Energy Increases Bone Formation during Arduous Military Training	Med Sci Sports Exerc	Excluded	Not exercise	
PACK6	32706442	Effects of eccentric training at long-muscle length on architectural and functional	Scand J Med Sci Sports	Included		

PACKS-RCT	PMID	Title	Journal/Book	Excluded or Included	Exclusion reason	Randomized ("YES" if selected)
		characteristics of the hamstrings				
PACK6	32711523	Long-term outcomes (2 and 3.5 years post-intervention) of the INFANT early childhood intervention to improve health behaviors and reduce obesity: cluster randomised controlled trial follow-up	Int J Behav Nutr Phys Act	Excluded	Wrong interventio n	
PACK6	32711957	Thermoregulatory responses to ice slurry ingestion during low and moderate intensity exercises with restrictive heat loss	J Sci Med Sport	Excluded	Not exercise	
PACK6	32718237	Intensity of statin treatment after acute coronary syndrome, residual risk, and its modification by alirocumab: insights from the ODYSSEY OUTCOMES trial	Eur J Prev Cardiol	Excluded	Non RCT	
PACK6	32722919	Effects of linagliptin on left ventricular DYsfunction in patients with type 2 DiAbetes and concentric left ventricular	Eur J Prev Cardiol	Excluded	Not exercise	

PACKS-RCT	PMID	Title	Journal/Book	Excluded or Included	Exclusion reason	Randomized ("YES" if selected)
		geometry: results of the DYDA 2 trial				
PACK6	32735109	Traditional and New Perspectives on Youth Cardiorespiratory Fitness	Med Sci Sports Exerc	Excluded	Non RCT	
PACK6	32735112	Bicarbonate Unlocks the Ergogenic Action of Ketone Monoester Intake in Endurance Exercise	Med Sci Sports Exerc	Excluded	Not exercise	
PACK6	32735114	Multidirectional Walking in Hematopoietic Stem Cell Transplant Patients	Med Sci Sports Exerc	Included		YES
PACK6	32773174	Postural stability during visual-based cognitive and motor dual-tasks after ACLR	J Sci Med Sport	Excluded	Non RCT	
PACK6	32798128	Interrogating cortical representations in elite athletes with persistent posterior thigh pain - New targets for intervention?	J Sci Med Sport	Excluded	Non RCT	
PACK7	31926869	'Maths on the move': Effectiveness of physically-active lessons for learning maths and increasing physical	J Sci Med Sport	Included		YES

PACKS-RCT	PMID	Title	Journal/Book	Excluded or Included	Exclusion reason	Randomized ("YES" if selected)
		activity in primary school students				
PACK7	32003063	Dose-response relationship of active commuting to work: Results of the GISMO study	Scand J Med Sci Sports	Excluded	Non RCT	
PACK7	32034943	Merging self-reported with technically sensed data for tracking mobility behavior in a naturalistic intervention study. Insights from the GISMO study	Scand J Med Sci Sports	Excluded	Non RCT	
PACK7	32049887	Velocity Loss as a Critical Variable Determining the Adaptations to Strength Training	Med Sci Sports Exerc	Included		YES
PACK7	32053547	Clinical versus Functional Reaction Time: Implications for Postconcussion Management	Med Sci Sports Exerc	Excluded	Non RCT	
PACK7	32079912	Caffeine Optimizes HIIT Benefits on Obesity-associated Metabolic Adversity in Women	Med Sci Sports Exerc	Excluded	Not exercise	
PACK7	32079913	Single-Joint and Whole-Body Movement Changes in Anterior Cruciate Ligament Athletes Returning to Sport	Med Sci Sports Exerc	Excluded	Non RCT	

PACKS-RCT	PMID	Title	Journal/Book	Excluded or Included	Exclusion reason	Randomized ("YES" if selected)
PACK7	32079914	Exercise Induces Different Molecular Responses in Trained and Untrained Human Muscle	Med Sci Sports Exerc	Excluded	Wrong interventio n	
PACK7	32079916	Leucine Supplementation Has No Further Effect on Training-induced Muscle Adaptations	Med Sci Sports Exerc	Excluded	Not exercise	
PACK7	32079917	A Machine Learning Approach to Assess Injury Risk in Elite Youth Football Players	Med Sci Sports Exerc	Excluded	Non RCT	
PACK7	32079920	Addition of an Alginate Hydrogel to a Carbohydrate Beverage Enhances Gastric Emptying	Med Sci Sports Exerc	Excluded	Not exercise	
PACK7	32079924	Lower-Limb Biomechanics in Football Players with and without Hip-related Pain	Med Sci Sports Exerc	Excluded	Non RCT	
PACK7	32102054	Is Individualization of Sodium Bicarbonate Ingestion Based on Time to Peak Necessary?	Med Sci Sports Exerc	Excluded	Not exercise	
PACK7	32102056	Heat Acclimation with Controlled Heart Rate: Influence of Hydration Status	Med Sci Sports Exerc	Excluded	Not exercise	

PACKS-RCT	PMID	Title	Journal/Book	Excluded or Included	Exclusion reason	Randomized ("YES" if selected)
PACK7	32102058	Hypoxic Exercise Training to Improve Exercise Capacity in Obese Individuals	Med Sci Sports Exerc	Included		
PACK7	32102062	An Animal Trial on the Optimal Time and Intensity of Exercise after Stroke	Med Sci Sports Exerc	Excluded	Non RCT	
PACK7	32246792	Effects of active commuting on health-related quality of life and sickness-related absence	Scand J Med Sci Sports	Included		
PACK7	32333707	Effects of active commuting to work for 12 months on cardiovascular risk factors and body composition	Scand J Med Sci Sports	Included		
PACK7	32335963	Effects of active commuting on cardiovascular risk factors: GISMO-a randomized controlled feasibility study	Scand J Med Sci Sports	Included		
PACK7	32361898	Sports Health During the SARS-Cov-2 Pandemic	Sports Med	Excluded	Non RCT	
PACK7	32680905	Correction: Exercise therapy for functional capacity in chronic diseases: an overview of meta-analyses of	Br J Sports Med	Excluded	Non RCT	

PACKS-RCT	PMID	Title	Journal/Book	Excluded or Included	Exclusion reason	Randomized ("YES" if selected)
		randomised controlled trials				
PACK7	32680906	Correction: Athletic groin pain (part 2): a prospective cohort study on the biomechanical evaluation of change of direction identifies three clusters of movement patterns	Br J Sports Med	Excluded	Non RCT	
PACK7	32738146	The interrater reliability of Dynamic Leap and Balance Test in Healthy and Chronic Ankle Instability	Scand J Med Sci Sports	Excluded	ong intervent	
PACK7	32746843	The association between recreational screen time and cancer risk: findings from the UK Biobank, a large prospective cohort study	Int J Behav Nutr Phys Act	Excluded	Non RCT	
PACK7	32746928	The effects of nudging and pricing on healthy food purchasing behavior in a virtual supermarket setting: a randomized experiment	Int J Behav Nutr Phys Act	Excluded	Not exercise	

PACKS-RCT	PMID	Title	Journal/Book	Excluded or Included	Exclusion reason	Randomized ("YES" if selected)
PACK7	32747406	Methods matter: dealing with low compliance in sports injury trials analyses using instrumental variable analysis	Br J Sports Med	Excluded	Non RCT	
PACK7	32749011	Action capability constrains visuo-motor complexity during planning and performance in on-sight climbing	Scand J Med Sci Sports	Excluded	Non RCT	
PACK7	32757327	Participation in sports/recreational activities and incidence of hypertension, diabetes, and obesity in adults	Scand J Med Sci Sports	Excluded	Non RCT	
PACK7	32769102	What makes a great clinical trial? Exploring the features of five important physiotherapy trials	Br J Sports Med	Excluded	Non RCT	
PACK7	32769811	Multifactorial Determinants of Running Injury Locations in 550 Injured Recreational Runners	Med Sci Sports Exerc	Excluded	Non RCT	

PACKS-RCT	PMID	Title	Journal/Book	Excluded or Included	Exclusion reason	Randomized ("YES" if selected)
PACK7	32771011	Scale-up of the Physical Activity 4 Everyone (PA4E1) intervention in secondary schools: 12-month implementation outcomes from a cluster randomized controlled trial	Int J Behav Nutr Phys Act	Included		YES
PACK7	32771018	Effect of novel technology-enabled multidimensional physical activity feedback in primary care patients at risk of chronic disease - the MIPACT study: a randomised controlled trial	Int J Behav Nutr Phys Act	Included		
PACK7	32776775	COMPOSITIONAL ASSOCIATIONS OF SLEEP AND ACTIVITIES WITHIN THE 24-H CYCLE WITH CARDIOMETABOLIC HEALTH MARKERS IN ADULTS	Med Sci Sports Exerc	Excluded	Non RCT	
PACK7	32783231	Variability in hemoglobin mass response to altitude training camps	Scand J Med Sci Sports	Excluded	Non RCT	
PACK7	32788296	Consent, capacity and compliance in concussion management: cave	Br J Sports Med	Excluded	Non RCT	

PACKS-RCT	PMID	Title	Journal/Book	Excluded or Included	Exclusion reason	Randomized ("YES" if selected)
		ergo medicus (let the doctor beware)				
PACK7	32789898	Exercise training-induced visceral fat loss in obese women: The role of training intensity and modality	Scand J Med Sci Sports	Included		
PACK7	32792317	Acute physiological responses with varying load or time under tension during a squat exercise: A randomized cross-over design	J Sci Med Sport	Excluded	Wrong interventio n	
PACK7	32796016	Does the Spraino low-friction shoe patch prevent lateral ankle sprain injury in indoor sports? A pilot randomised controlled trial with 510 participants with previous ankle injuries	Br J Sports Med	Excluded	Not exercise	
PACK7	32796255	Effects of Eight Weeks of 16: 8 Time-restricted Eating in Male Middle- and Long-Distance Runners	Med Sci Sports Exerc	Excluded	Not exercise	
PACK7	32804901	Hemodynamic Response to Isometric Handgrip Exercise in Adults	Med Sci Sports Exerc	Excluded	Non RCT	

PACKS-RCT	PMID	Title	Journal/Book	Excluded or Included	Exclusion reason	Randomized ("YES" if selected)
		with Intellectual Disability				
PACK7	32807194	Effects of the KEIGAAF intervention on the BMI z-score and energy balance-related behaviors of primary school-aged children	Int J Behav Nutr Phys Act	Excluded	Non RCT	
PACK7	32816787	Optimising outcomes of exercise and corticosteroid injection in patients with subacromial pain (impingement) syndrome: a factorial randomised trial	Br J Sports Med	Included		YES
PACK7	32826226	Association of physical activity with risk of hepatobiliary diseases in China: a prospective cohort study of 0.5 million people	Br J Sports Med	Excluded	Non RCT	
PACK7	32826630	Aerobic Fitness Is Related to Myocardial Fibrosis Post-Anthracycline Therapy	Med Sci Sports Exerc	Excluded	Non RCT	
PACK7	32826631	Impact of 6-Month Exercise Training on Neurovascular Function in Spinal Cord Injury	Med Sci Sports Exerc	Excluded	Non RCT	

PACKS-RCT	PMID	Title	Journal/Book	Excluded or Included	Exclusion reason	Randomized ("YES" if selected)
PACK7	32826632	Novel CPET Reference Values in Healthy Adults: Associations with Physical Activity	Med Sci Sports Exerc	Excluded	Non RCT	
PACK7	32826633	Effects of BMI on Walking Speed and Gait Biomechanics following ACL Reconstruction	Med Sci Sports Exerc	Excluded	Non RCT	
PACK7	32826635	Blood Flow Restriction Training Blunts Chronic Kidney Disease Progression in Humans	Med Sci Sports Exerc	Included		YES
PACK7	32826636	Long-Term and Acute Benefits of Reduced Sitting on Vascular Flow and Function	Med Sci Sports Exerc	Excluded	Non RCT	
PACK7	32826637	Whole-Body Vibration Stimulates Microvascular Blood Flow in Skeletal Muscle	Med Sci Sports Exerc	Excluded	Wrong interventio n	
PACK7	32839106	Injuries according to the percentage of adult height in an elite soccer academy	J Sci Med Sport	Excluded	Non RCT	
PACK7	32839107	Negative association between injuries and team success in professional cricket: A 9-year prospective cohort analysis	J Sci Med Sport	Excluded	Non RCT	

PACKS-RCT	PMID	Title	Journal/Book	Excluded or Included	Exclusion reason	Randomized ("YES" if selected)
PACK7	32846028	Epidemiology of all-complaint injuries in youth basketball	Scand J Med Sci Sports	Excluded	Non RCT	
PACK7	32847731	Probiotic supplementation elicits favourable changes in muscle soreness and sleep quality in rugby players	J Sci Med Sport	Excluded	Wrong interventio n	
PACK7	32847812	Use of the Sport Concussion Assessment Tool 5 (SCAT5) in professional hockey, part 2: which components differentiate concussed and non-concussed players?	Br J Sports Med	Excluded	Non RCT	
PACK7	32854151	Stretch reflex changes in ACL-deficient individuals and healthy controls during normal and surprise landings	Scand J Med Sci Sports	Excluded	Non RCT	
PACK7	32854153	Integrating high-intensity interval training into the workplace: The Work-HIIT pilot RCT	Scand J Med Sci Sports	Excluded	Non RCT	

PACKS-RCT	PMID	Title	Journal/Book	Excluded or Included	Exclusion reason	Randomized ("YES" if selected)
PACK7	32855201	Arthroscopic partial meniscectomy for a degenerative meniscus tear: a 5 year follow-up of the placebo-surgery controlled FIDELITY (Finnish Degenerative Meniscus Lesion Study) trial	Br J Sports Med	Excluded	Wrong interventio n	
PACK7	32859522	Identifying ActiGraph non-wear time in pregnant women with overweight or obesity	J Sci Med Sport	Excluded	Wrong interventio n	
PACK7	32868201	In-competition injuries and performance success in combined events during major international athletics championships	J Sci Med Sport	Excluded	Non RCT	
PACK7	32868315	Injuries and illnesses among competitive Norwegian rhythmic gymnasts during preseason: a prospective cohort study of prevalence, incidence and risk factors	Br J Sports Med	Excluded	Non RCT	

PACKS-RCT	PMID	Title	Journal/Book	Excluded or Included	Exclusion reason	Randomized ("YES" if selected)
PACK7	32868580	The Effect of Blood Ketone Concentration and Exercise Intensity on Exogenous Ketone Oxidation Rates in Athletes	Med Sci Sports Exerc	Excluded	Wrong interventio n	
PACK7	32881770	Proximal Aortic Compliance in Young Male Endurance Athletes: An MRI Study	Med Sci Sports Exerc	Excluded	Wrong interventio n	
PACK7	32896459	What factors are associated with physical activity promotion in the podiatry setting? A cross-sectional study	J Sci Med Sport	Excluded	Non RCT	
pack 8	32998848	Distribution of concurrent training sessions does not impact endurance adaptation	J Sci Med Sport	Included		YES
pack 8	32991345	Investigating the Matching Relationship between Physical Exercise and Stereotypic Behavior in Children with Autism	Med Sci Sports Exerc	Excluded	Non RCT	

PACKS-RCT	PMID	Title	Journal/Book	Excluded or Included	Exclusion reason	Randomized ("YES" if selected)
pack 8	32988932	It is never too late to start: adherence to physical activity recommendations for 11-22 years and risk of all-cause and cardiovascular disease mortality. The HUNT Study	Br J Sports Med	Excluded	Non RCT	
pack 8	32988930	Efficacy of heel lifts versus calf muscle eccentric exercise for mid-portion Achilles tendinopathy (HEALTHY): a randomised trial	Br J Sports Med	Excluded		YES
pack 8	32972979	Successful return to professional men's football (soccer) competition after the COVID-19 shutdown: a cohort study in the German Bundesliga	Br J Sports Med	Excluded	Non RCT	
pack 8	32969540	Minute oscillation stretching: a novel modality for reducing musculotendinous stiffness and maintaining muscle strength	Scand J Med Sci Sports	Excluded	Non RCT	
pack 8	32969535	Adaptation to a ketogenic diet modulates adaptive and mucosal immune markers in trained	Scand J Med Sci Sports	Excluded	Non RCT	

PACKS-RCT	PMID	Title	Journal/Book	Excluded or Included	Exclusion reason	Randomized ("YES" if selected)
		male endurance athletes				
pack 8	32969531	Return to training in the COVID-19 era: The physiological effects of face masks during exercise	Scand J Med Sci Sports	Excluded	Wrong interventio n	
pack 8	32967854	Return to sport for North American professional sport leagues in the context of COVID-19	Br J Sports Med	Excluded	Non RCT	
pack 8	32962724	A whole family-based physical activity promotion intervention: findings from the families reporting every step to health (FRESH) pilot randomised controlled trial	Int J Behav Nutr Phys Act	Excluded	Wrong interventio n	
pack 8	32958468	Patients use fewer analgesics following supervised exercise therapy and patient education: an observational study of 16 499 patients with knee or hip osteoarthritis	Br J Sports Med	Excluded	Non RCT	

PACKS-RCT	PMID	Title	Journal/Book	Excluded or Included	Exclusion reason	Randomized ("YES" if selected)
pack 8	32958377	Effect of motor imagery on enjoyment in knee-injury prevention and rehabilitation training: A randomized crossover study	J Sci Med Sport	Excluded	Wrong interventio n	
pack 8	32951977	Epidemiology of injuries in Australian junior rugby league players	J Sci Med Sport	Excluded	Non RCT	
pack 8	32949027	Role of CaMKII and sarcolipin in muscle adaptations to strength training with different levels of fatigue in the set	Scand J Med Sci Sports	Excluded	Wrong interventio n	
pack 8	32945566	Associations of sedentary behavior bouts with community-dwellin g older adults' physical function	Scand J Med Sci Sports	Excluded	Non RCT	
pack 8	32939858	Gait biofeedback and impairment-based rehabilitation for chronic ankle instability	Scand J Med Sci Sports	Included		YES
pack 8	32928654	Exercise & Sports Science Australia (ESSA) position statement on exercise and chronic obstructive pulmonary disease	J Sci Med Sport	Excluded	Non RCT	

PACKS-RCT	PMID	Title	Journal/Book	Excluded or Included	Exclusion reason	Randomized ("YES" if selected)
pack 8	32928228	The effects of an abrupt increase in taxes on candy and soda in Norway: an observational study of retail sales	Int J Behav Nutr Phys Act	Excluded	Non RCT	
pack 8	32925495	A Low-Calorie Diet with or without Exercise Reduces Postprandial Aortic Waveform in Females with Obesity	Med Sci Sports Exerc	Excluded	Wrong interventio n	
pack 8	32925494	Interrupting Prolonged Sitting and Endothelial Function in Polycystic Ovary Syndrome	Med Sci Sports Exerc	Excluded	Wrong interventio n	
pack 8	32917672	An intervention to optimise coach-created motivational climates and reduce athlete willingness to dope (CoachMADE): a three-country cluster randomised controlled trial	Br J Sports Med	Excluded	Wrong interventio n	
pack 8	32917671	Prospective study of the association between sport-related concussion and brain morphometry (3T-MRI) in collegiate athletes: study from the	Br J Sports Med	Excluded	Non RCT	

PACKS-RCT	PMID	Title	Journal/Book	Excluded or Included	Exclusion reason	Randomized ("YES" if selected)
		NCAA-DoD CARE Consortium				
pack 8	32912260	Leveraging family dynamics to increase the effectiveness of incentives for physical activity: the FIT-FAM randomized controlled trial	Int J Behav Nutr Phys Act	Excluded	Wrong interventio n	
pack 8	32910096	Cricket Fast Bowling Technique and Lumbar Bone Stress Injury	Med Sci Sports Exerc	Excluded	Non RCT	
pack 8	32910094	Inflammation Mediates Exercise Effects on Fatigue in Patients with Breast Cancer	Med Sci Sports Exerc	Included		YES
pack 8	32901996	Prolonging the duration of cooling does not enhance recovery following a marathon	Scand J Med Sci Sports	Excluded	Wrong interventio n	
pack 8	32897568	Contralateral effects of eccentric resistance training on immobilized arm	Scand J Med Sci Sports	Excluded	Wrong interventio n	
pack 8	32897526	Effects of Vertically and Horizontally Orientated Plyometric Training on Physical Performance: A Meta-analytical Comparison	Sports Med	Excluded	Non RCT	

PACKS-RCT	PMID	Title	Journal/Book	Excluded or Included	Exclusion reason	Randomized ("YES" if selected)
pack 8	32881097	Elite female athletes' experiences and perceptions of the menstrual cycle on training and sport performance	Scand J Med Sci Sports	Excluded	Non RCT	
pack 8	32881054	Acute metabolic responses after continuous or interval exercise in post-menopausal women with overweight or obesity	Scand J Med Sci Sports	Included		YES
pack 8	32878870	Cardiorespiratory considerations for return-to-play in elite athletes after COVID-19 infection: a practical guide for sport and exercise medicine physicians	Br J Sports Med	Excluded	Non RCT	
pack 8	32878869	Custom insoles versus sham and GP-led usual care in patients with plantar heel pain: results of the STAP-study - a randomised controlled trial	Br J Sports Med	Excluded	Wrong interventio n	
pack 8	32816837	Correction: Match injury incidence during the Super Rugby tournament is high: a prospective cohort study over five	Br J Sports Med	Excluded	Non RCT	

PACKS-RCT	PMID	Title	Journal/Book	Excluded or Included	Exclusion reason	Randomized ("YES" if selected)
		seasons involving 93 641 player-hours				
pack 8	32265218	Towards a better understanding of the 'physical activity paradox': the need for a research agenda	Br J Sports Med	Excluded	Non RCT	
pack 8	32220853	Mouthguards should be worn in contact sports	Br J Sports Med	Excluded	Non RCT	
pack 8	32217523	Ski racers' understanding of sports-related concussion and its management: are contemporary findings and clinical recommendations reaching the target audience, the racers themselves?	Br J Sports Med	Excluded	Non RCT	
pack 8	31694812	Infographic running myth: static stretching reduces injury risk in runners	Br J Sports Med	Excluded	Non RCT	
Pack 9	31818846	How to recognise sudden cardiac arrest on the pitch	Br J Sports Med	Excluded	Non RCT	
Pack 9	31840535	Free cholesterol transfer to high-density lipoprotein (HDL) upon triglyceride lipolysis underlies the U-shape relationship between	Eur J Prev Cardiol	Excluded	Non RCT	

PACKS-RCT	PMID	Title	Journal/Book	Excluded or Included	Exclusion reason	Randomized ("YES" if selected)
		HDL-cholesterol and cardiovascular disease				
Pack 9	31857336	Maximising and evaluating the uptake, use and impact of golf and health studies	Br J Sports Med	Excluded	Non RCT	
Pack 9	32139368	Methods matter: exploring the 'too much, too soon' theory, part 1: causal questions in sports injury research	Br J Sports Med	Excluded	Non RCT	
Pack 9	32229771	Protein Supplementation and Resistance Training in Childhood Cancer Survivors	Med Sci Sports Exerc	Excluded	Not exercise	
Pack 9	32251253	No Sex Difference in Mental Fatigue Effect on High-Level Runners' Aerobic Performance	Med Sci Sports Exerc	Excluded	Non RCT	
Pack 9	32251254	Using TENS to Enhance Therapeutic Exercise in Individuals with Knee Osteoarthritis	Med Sci Sports Exerc	Included		YES
Pack 9	32251255	Prevention of Decompression Sickness by Novel Artificial Oxygen Carriers	Med Sci Sports Exerc	Excluded	Non RCT	

PACKS-RCT	PMID	Title	Journal/Book	Excluded or Included	Exclusion reason	Randomized ("YES" if selected)
Pack 9	32265217	Infographic. Diagnosis, prevention and treatment of common lower extremity muscle injuries in sport-grading the evidence: a statement paper commissioned by the Danish Society of Sports Physical Therapy (DSSF)	Br J Sports Med	Excluded	Non RCT	
Pack 9	32282451	Muscle Oxidative Capacity Is Reduced in Both Upper and Lower Limbs in COPD	Med Sci Sports Exerc	Excluded	Non RCT	
Pack 9	32301853	Effect of Strength Training on Glycemic Control and Adiponectin in Diabetic Children	Med Sci Sports Exerc	Included		YES
Pack 9	32301854	Adaptive Control of Dynamic Balance across the Adult Lifespan	Med Sci Sports Exerc	Excluded	Non RCT	
Pack 9	32327485	Strong and stringent hamstring strain science: trials and error!	Br J Sports Med	Excluded	Non RCT	
Pack 9	32496738	Microvascular Function Is Impaired after Short-Term Immobilization in Healthy Men	Med Sci Sports Exerc	Excluded	Non RCT	

PACKS-RCT	PMID	Title	Journal/Book	Excluded or Included	Exclusion reason	Randomized ("YES" if selected)
Pack 9	32546560	Position statement of the Royal Spanish Football Federation for the resumption of football activities after the COVID-19 pandemic (June 2020)	Br J Sports Med	Excluded	Non RCT	
Pack 9	32661840	Cognitive Fatigue Effects on Physical Performance: The Role of Interoception	Sports Med	Excluded	Non RCT	
Pack 9	32936593	Plantar Flexor Function in Adults with and without Prader-Willi Syndrome	Med Sci Sports Exerc	Excluded	Non RCT	
Pack 9	32936595	Not Just for Joints: The Associations of Moderate-to-Vigorous Physical Activity and Sedentary Behavior with Brain Cortical Thickness	Med Sci Sports Exerc	Excluded	Wrong interventio n	
Pack 9	32936596	Burn Injury Does Not Exacerbate Heat Strain during Exercise while Wearing Body Armor	Med Sci Sports Exerc	Excluded	Wrong interventio n	
Pack 9	33009195	Weight Loss for Obese Prostate Cancer Patients on Androgen Deprivation Therapy	Med Sci Sports Exerc	Excluded	Wrong interventio n	

PACKS-RCT	PMID	Title	Journal/Book	Excluded or Included	Exclusion reason	Randomized ("YES" if selected)
Pack 9	33009196	The Muscle Morphology of Elite Sprint Running	Med Sci Sports Exerc	Excluded	Non RCT	
Pack 9	33009197	Greater Hamstrings Muscle Hypertrophy but Similar Damage Protection after Training at Long versus Short Muscle Lengths	Med Sci Sports Exerc	Excluded	Wrong interventio n	
Pack 9	33017351	Measuring the Exercise Component of Energy Availability during Arduous Training in Women	Med Sci Sports Exerc	Excluded	Non RCT	
Pack 9	33017352	A Genome-wide Association Study for Concussion Risk	Med Sci Sports Exerc	Excluded	Non RCT	
Pack 9	33020137	Subacromial decompression versus diagnostic arthroscopy for shoulder impingement: a 5-year follow-up of a randomised, placebo surgery controlled clinical trial	Br J Sports Med	Excluded	Wrong interventio n	
Pack 9	33028335	Which behaviour change techniques are effective to promote physical activity and reduce sedentary behaviour in adults: a factorial randomized trial of	Int J Behav Nutr Phys Act	Excluded	Wrong interventio n	

PACKS-RCT	PMID	Title	Journal/Book	Excluded or Included	Exclusion reason	Randomized ("YES" if selected)
		an e- and m-health intervention				
Pack 9	33028585	Infographic. Does foot mobility affect the outcome in the management of patellofemoral pain with foot orthoses versus hip exercises? A randomised clinical trial	Br J Sports Med	Excluded	Non RCT	
Pack 9	33032991	Managing head injury risks in competitive skateboarding: what do we know?	Br J Sports Med	Excluded	Non RCT	
Pack 9	33036885	Prevalence of fundamental movement skill proficiency among Iranian children aged 2.5-14 years	J Sci Med Sport	Excluded	Non RCT	
Pack 9	33036995	Does load management using the acute:chronic workload ratio prevent health problems? A cluster randomised trial of 482 elite youth footballers of both sexes	Br J Sports Med	Included		YES
Pack 9	33038018	Training load but not fatigue affects cross-education of maximal voluntary force	Scand J Med Sci Sports	Included		YES

PACKS-RCT	PMID	Title	Journal/Book	Excluded or Included	Exclusion reason	Randomized ("YES" if selected)
Pack 9	33038020	Positive and negative side effects of androgen abuse. The HAARLEM study: A one-year prospective cohort study in 100 men	Scand J Med Sci Sports	Excluded	Non RCT	
Pack 9	33038026	Estimation of the second ventilatory threshold through ventricular repolarization profile analysis	Scand J Med Sci Sports	Excluded	Non RCT	
Pack 9	33038051	Effects of a resistance training program in kidney transplant recipients: A randomized controlled trial	Scand J Med Sci Sports	Included		
Pack 9	33044435	Increased Duration of Exercise Decreases Rate of Nonresponse to Exercise but May Not Decrease Risk for Cancer Mortality	Med Sci Sports Exerc	Included		
Pack 9	33044437	Carbohydrate Supplementation and the Influence of Breakfast on Fuel Use in Hypoxia	Med Sci Sports Exerc	Excluded	Wrong interventio n	
Pack 9	33044439	The Effects of Fire Academy Training and Probationary Firefighter Status on Select Basic	Med Sci Sports Exerc	Excluded	Non RCT	

PACKS-RCT	PMID	Title	Journal/Book	Excluded or Included	Exclusion reason	Randomized ("YES" if selected)
		Health and Fitness Measurements				
Pack 9	33044440	Caffeine Exacerbates Hyperventilation and Reductions in Cerebral Blood Flow in Physically Fit Males Exercising in the Heat	Med Sci Sports Exerc	Excluded	Wrong interventio n	
Pack 9	33044441	A Single Bout of Premeal Resistance Exercise Improves Postprandial Glucose Metabolism in Obese Men with Prediabetes	Med Sci Sports Exerc	Excluded	Wrong interventio n	
Pack 9	33055132	Infographic. Does the Spraino low-friction shoe patch prevent lateral ankle sprain injury in indoor sports? A 510-participant pilot RCT	Br J Sports Med	Excluded	Non RCT	
Pack 9	33055134	Diagnosing Achilles tendinopathy is like delicious spaghetti carbonara: it is all about key ingredients, but not all chefs use the same recipe	Br J Sports Med	Excluded	Non RCT	

PACKS-RCT	PMID	Title	Journal/Book	Excluded or Included	Exclusion reason	Randomized ("YES" if selected)
Pack 9	33060548	Short- and Long-Term Stroboscopic Training Effects on Visuomotor Performance in Elite Youth Sports. Part 1: Reaction and Behavior	Med Sci Sports Exerc	Excluded	Wrong interventio n	
Pack 9	33060549	Short- and Long-Term Stroboscopic Training Effects on Visuomotor Performance in Elite Youth Sports. Part 2: Brain-Behavior Mechanisms	Med Sci Sports Exerc	Excluded	Wrong interventio n	
Pack 9	33063268	Associations of Objectively Measured Physical Activity and Sedentary Time with the Risk of Stroke, Myocardial Infarction or All-Cause Mortality in 70-Year-Old Men and Women: A Prospective Cohort Study	Sports Med	Excluded	Non RCT	
Pack 9	33065594	Diurnal versus Nocturnal Exercise-Impact on the Gastrointestinal Tract	Med Sci Sports Exerc	Excluded	Wrong interventio n	

PACKS-RCT	PMID	Title	Journal/Book	Excluded or Included	Exclusion reason	Randomized ("YES" if selected)
Pack 9	33065595	Heat Acclimation Improves Heat Tolerance Test Specificity in a Criteria-dependent Manner	Med Sci Sports Exerc	Excluded	Wrong interventio n	
Pack 9	33071199	The impact of the mechanical whole-body vibration experienced during military land transit on the physical attributes underpinning dismounted combatant physical performance: A randomised controlled trial	J Sci Med Sport	Excluded	Wrong interventio n	
Pack 9	33077401	The prediction of thriving in elite sport: A prospective examination of the role of psychological need satisfaction, challenge appraisal, and salivary biomarkers	J Sci Med Sport	Excluded	Non RCT	
Pack 9	33079394	Crank length alters kinematics and kinetics, yet not the economy of recumbent handcyclists at constant handgrip speeds	Scand J Med Sci Sports	Excluded	Non RCT	

PACKS-RCT	PMID	Title	Journal/Book	Excluded or Included	Exclusion reason	Randomized ("YES" if selected)
Pack 9	33105384	Reliability and Validity of Self-reported Muscle-strengthening Exercise in the Cancer Prevention Study-3	Med Sci Sports Exerc	Excluded	Non RCT	
Pack 9	33105385	Exercise Intolerance in Cystic Fibrosis: Importance of Skeletal Muscle	Med Sci Sports Exerc	Excluded	Non RCT	
Pack 9	33105387	Daily Step Count and Postprandial Fat Metabolism	Med Sci Sports Exerc	Excluded	Wrong intervention	
Pack 9	33105388	An Ethanolic Extract of Artemisia dracunculus L. Enhances the Metabolic Benefits of Exercise in Diet-induced Obese Mice	Med Sci Sports Exerc	Excluded	Animals	
Pack 9	33105390	Blood Flow Restriction Combined with Electrical Stimulation Attenuates Thigh Muscle Disuse Atrophy	Med Sci Sports Exerc	Excluded	Wrong intervention	
Pack 9	33109190	Efficacy of the 'Stand and Move at Work' multicomponent workplace intervention to reduce sedentary time and improve cardiometabolic	Int J Behav Nutr Phys Act	Included		YES

PACKS-RCT	PMID	Title	Journal/Book	Excluded or Included	Exclusion reason	Randomized ("YES" if selected)
		risk: a group randomized clinical trial				
Pack 9	33113211	Strength training restores force-generating capacity in patients with schizophrenia	Scand J Med Sci Sports	Included		
Pack 9	33113253	Effects of including sprints during prolonged cycling on hormonal and muscular responses and recovery in elite cyclists	Scand J Med Sci Sports	Excluded	Wrong interventio n	
Pack 10	31787023	Non-linear is not superior to linear aerobic training periodization in coronary heart disease patients	Eur J Prev Cardiol	Excluded	Duplicate pos elegibility	
Pack 10	31852300	Predictors of pre-rehabilitation exercise capacity in elderly European cardiac patients - The EU-CaRE study	Eur J Prev Cardiol	Excluded	Non RCT	
Pack 10	32471813	Physical activity and health in Chinese children and adolescents: expert consensus statement (2020)	Br J Sports Med	Excluded	Non RCT	
Pack 10	32661127	The Australian Institute of Sport (AIS) and National Eating Disorders Collaboration (NEDC) position statement on	Br J Sports Med	Excluded	Non RCT	

PACKS-RCT	PMID	Title	Journal/Book	Excluded or Included	Exclusion reason	Randomized ("YES" if selected)
		disordered eating in high performance sport				
Pack 10	32699001	Are questionable research practices facilitating new discoveries in sport and exercise medicine? The proportion of supported hypotheses is implausibly high	Br J Sports Med	Excluded	Non RCT	
Pack 10	32788295	Infographic. Football-specific strategies to reduce COVID-19 transmission	Br J Sports Med	Excluded	Non RCT	
Pack 10	33064403	Preseason Screen Cannot Predict Injury over Three Years of College Football	Med Sci Sports Exerc	Excluded	Non RCT	
Pack 10	33064404	Effects of Cognitive Training and Exergaming in Pediatric Cancer Survivors-A Randomized Clinical Trial	Med Sci Sports Exerc	Included		
Pack 10	33064408	Sex and Sport Differences in College Lacrosse and Soccer Head Impact Biomechanics	Med Sci Sports Exerc	Excluded	Non RCT	
Pack 10	33064410	Is There a Link between Stress and Cognition, and Capacity to	Med Sci Sports Exerc	Excluded	Non RCT	

PACKS-RCT	PMID	Title	Journal/Book	Excluded or Included	Exclusion reason	Randomized ("YES" if selected)
		Execute Motor Skill?				
Pack 10	33064411	Respiratory Muscle Fatigue Alters Cycling Performance and Locomotor Muscle Fatigue	Med Sci Sports Exerc	Excluded	Non RCT	
Pack 10	33064412	Exercise Training Improves Microvascular Function in Burn Injury Survivors	Med Sci Sports Exerc	Excluded	Non RCT	
Pack 10	33064413	Synergetic Effect of NO Precursor Supplementation and Exercise Training	Med Sci Sports Exerc	Excluded	Wrong interventio n	
Pack 10	33064415	Exercise for Weight Loss: Further Evaluating Energy Compensation with Exercise	Med Sci Sports Exerc	Included		
Pack 10	33144346	Are web-based personally tailored physical activity videos more effective than personally tailored text-based interventions? Results from the three-arm randomised controlled TaylorActive trial	Br J Sports Med	Included		
Pack 10	33144348	Injury characteristics in male youth athletics: a five-season	Br J Sports Med	Excluded	Non RCT	

PACKS-RCT	PMID	Title	Journal/Book	Excluded or Included	Exclusion reason	Randomized ("YES" if selected)
		prospective study in a full-time sports academy				
Pack 10	33144350	i-CONTENT tool for assessing therapeutic quality of exercise programs employed in randomised clinical trials	Br J Sports Med	Excluded	Non RCT	
Pack 10	33148972	Kinetics of Muscle Carnosine Decay after β-alanine Supplementation: A 16-Week Washout Study	Med Sci Sports Exerc	Excluded	Non RCT	
Pack 10	33155295	Impact of exercise training and supplemental oxygen on submaximal exercise performance in patients with COPD	Scand J Med Sci Sports	Excluded	Wrong interventio n	
Pack 10	33160856	Neurocognitive changes associated with concussion in elite cricket players are distinct from changes due to post-match with no head impact	J Sci Med Sport	Excluded	Non RCT	
Pack 10	33162329	Branched-chain amino acid supplementation improves cycling performance in untrained cyclists	J Sci Med Sport	Excluded	Wrong interventio n	

PACKS-RCT	PMID	Title	Journal/Book	Excluded or Included	Exclusion reason	Randomized ("YES" if selected)
Pack 10	33167687	CORRIGENDUM: The effects and costs of home-based rehabilitation for heart failure with reduced ejection fraction: The REACH-HF multicentre randomized controlled trial	Eur J Prev Cardiol	Excluded	Non RCT	
Pack 10	33167995	Prospective association between organic food consumption and the risk of type 2 diabetes: findings from the NutriNet-Santé cohort study	Int J Behav Nutr Phys Act	Excluded	Non RCT	
Pack 10	33176018	Effects of cryotherapy on the regeneration process and muscular mechanical properties after lacerative injury model	Scand J Med Sci Sports	Excluded	Non RCT	
Pack 10	33184114	Aetiology and incidence of sudden cardiac arrest and death in young competitive athletes in the USA: a 4-year prospective study	Br J Sports Med	Excluded	Non RCT	

PACKS-RCT	PMID	Title	Journal/Book	Excluded or Included	Exclusion reason	Randomized ("YES" if selected)
Pack 10	33185897	Combination of resistance and aerobic exercise for six months improves bone mass and physical function in HIV infected individuals: A randomized controlled trial	Scand J Med Sci Sports	Included		YES
Pack 10	33187880	Do people with unilateral mid-portion Achilles tendinopathy who participate in running-related physical activity exhibit a meaningful conditioned pain modulation (CPM) effect: a pilot study	J Sci Med Sport	Excluded	Non RCT	
Pack 10	33189596	Running behavior and symptoms of respiratory tract infection during the COVID-19 pandemic: A large prospective Dutch cohort study	J Sci Med Sport	Excluded	Non RCT	
Pack 10	33196605	Exogenous Ketosis Impairs 30-min Time-Trial Performance Independent of Bicarbonate Supplementation	Med Sci Sports Exerc	Excluded	Wrong interventio n	

PACKS-RCT	PMID	Title	Journal/Book	Excluded or Included	Exclusion reason	Randomized ("YES" if selected)
Pack 10	33196606	Acute Running and Coronary Heart Disease Risk Markers in Male Cigarette Smokers and Nonsmokers: A Randomized Crossover Trial	Med Sci Sports Exerc	Included		YES
Pack 10	33198790	Calibration of the food parenting practice (FPP) item bank: tools for improving the measurement of food parenting practices of parents of 5-12-year-old children	Int J Behav Nutr Phys Act	Excluded	Non RCT	
Pack 10	33199359	Injury incidence and burden in a youth elite football academy: a four-season prospective study of 551 players aged from under 9 to under 19 years	Br J Sports Med	Excluded	Non RCT	
Pack 10	33199360	Prevalence and frequency of menstrual cycle symptoms are associated with availability to train and compete: a study of 6812 exercising women recruited using the Strava exercise app	Br J Sports Med	Excluded	Non RCT	

PACKS-RCT	PMID	Title	Journal/Book	Excluded or Included	Exclusion reason	Randomized ("YES" if selected)
Pack 10	33202068	Are PROMs used adequately in sports research? An analysis of 54 randomized controlled trials with PROMs as endpoint	Scand J Med Sci Sports	Excluded	Non RCT	
Pack 10	33208166	Adding team-based financial incentives to the Carrot Rewards physical activity app increases daily step count on a population scale: a 24-week matched case control study	Int J Behav Nutr Phys Act	Excluded	Non RCT	
Pack 10	33213459	Objective understanding of the Nutri-score front-of-pack label by European consumers and its effect on food choices: an online experimental study	Int J Behav Nutr Phys Act	Excluded	Non RCT	
Pack 10	33213465	The reciprocal effects of physical activity and happiness in adolescents	Int J Behav Nutr Phys Act	Excluded	Wrong interventio n	
Pack 10	33218873	Concurrent brain endurance training improves endurance exercise performance	J Sci Med Sport	Excluded	Mixed RCT	

PACKS-RCT	PMID	Title	Journal/Book	Excluded or Included	Exclusion reason	Randomized ("YES" if selected)
Pack 10	33219113	Injury incidence, severity and profile in Olympic combat sports: a comparative analysis of 7712 athlete exposures from three consecutive Olympic Games	Br J Sports Med	Excluded	Non RCT	
Pack 10	33219115	Effectiveness of progressive tendon-loading exercise therapy in patients with patellar tendinopathy: a randomised clinical trial	Br J Sports Med	Included		
Pack 10	33222326	Minimally Important Change and Smallest Detectable Change of the OSTRC Questionnaire in Half- and Full-Marathon Runners	Scand J Med Sci Sports	Excluded	Non RCT	
Pack 10	33226660	Effects of interrupting sitting with different activity bouts on postprandial lipemia: A randomized crossover trial	Scand J Med Sci Sports	Excluded	ong intervent	

PACKS-RCT	PMID	Title	Journal/Book	Excluded or Included	Exclusion reason	Randomized ("YES" if selected)
Pack 10	33243246	The effect of a school-based intervention on physical activity, cardiorespiratory fitness and muscle strength: the School in Motion cluster randomized trial	Int J Behav Nutr Phys Act	Included		YES
Pack 10	33243595	Training injury incidence in an amateur women's rugby union team in New Zealand over two consecutive seasons	J Sci Med Sport	Excluded	Non RCT	
Pack 10	33247001	Randomised controlled trial of an augmented exercise referral scheme using web-based behavioural support for inactive adults with chronic health conditions: the e-coachER trial	Br J Sports Med	Included		YES
Pack 10	33247965	Strength development and non-contact lower limb injury in academy footballers across ages groups	Scand J Med Sci Sports	Excluded	Non RCT	

PACKS-RCT	PMID	Title	Journal/Book	Excluded or Included	Exclusion reason	Randomized ("YES" if selected)
Pack 10	33256737	Understanding the influence of physical resources and social supports on primary food providers' snack food provision: a discrete choice experiment	Int J Behav Nutr Phys Act	Excluded	Non RCT	
Pack 10	33256753	A physical activity coaching intervention can improve and maintain physical activity and health-related outcomes in adult ambulatory hospital patients: the Healthy4U-2 randomised controlled trial	Int J Behav Nutr Phys Act	Included		YES
Pack 10	33257175	Epidemiology of skydiving-related deaths and injuries: A 10-years prospective study of 6.2 million jumps between 2010 and 2019 in France	J Sci Med Sport	Excluded	Non RCT	
Pack 10	33262041	Household illness is the strongest predictor of upper respiratory tract symptom risk in elite rugby union players	J Sci Med Sport	Excluded	Non RCT	

PACKS-RCT	PMID	Title	Journal/Book	Excluded or Included	Exclusion reason	Randomized ("YES" if selected)
PACK 11	33394902	Bone Microarchitecture and Strength Adaptation to Physical Activity: A Within-Subject Controlled, HRpQCT Study	Med Sci Sports Exerc	Excluded	Non RCT	
PACK 11	33394901	Both Traditional and Stair Climbing-based HIIT Cardiac Rehabilitation Induce Beneficial Muscle Adaptations	Med Sci Sports Exerc	Included		YES
PACK 11	33386238	The effect of mental fatigue on the performance of Australian football specific skills amongst amateur athletes	J Sci Med Sport	Excluded	Wrong interventio n	
PACK 11	33382128	Applying a holistic hamstring injury prevention approach in elite football: 12 seasons, single club study	Scand J Med Sci Sports	Excluded	Non RCT	
PACK 11	33380379	Absence of dietary control precludes solid conclusions for sport nutrition trials	J Sci Med Sport	Excluded	Non RCT	

PACKS-RCT	PMID	Title	Journal/Book	Excluded or Included	Exclusion reason	Randomized ("YES" if selected)
PACK 11	33376076	Lower preseason reactive strength index scores are associated with injury in female collegiate volleyball players but not male collegiate basketball players	J Sci Med Sport	Excluded	Non RCT	
PACK 11	33361278	Interassociation consensus recommendations for pitch-side emergency care and personal protective equipment for elite sport during the COVID-19 pandemic	Br J Sports Med	Excluded	Non RCT	
PACK 11	33361136	Acute glycaemic management before, during and after exercise for cardiac rehabilitation participants with diabetes mellitus: a joint statement of the British and Canadian Associations of Cardiovascular Prevention and Rehabilitation, the International Council for Cardiovascular Prevention and Rehabilitation and	Br J Sports Med	Excluded	Wrong interventio n	

PACKS-RCT	PMID	Title	Journal/Book	Excluded or Included	Exclusion reason	Randomized ("YES" if selected)
		the British Association of Sport and Exercise Sciences				
PACK 11	33361135	FIFA Sudden Death Registry (FIFA-SDR): a prospective, observational study of sudden death in worldwide football from 2014 to 2018	Br J Sports Med	Excluded	Non RCT	
PACK 11	33361134	Injury patterns differ with age in male youth football: a four-season prospective study of 1111 time-loss injuries in an elite national academy	Br J Sports Med	Excluded	Non RCT	
PACK 11	33358586	Trunk endurance, posterior chain flexibility, and previous history of musculoskeletal pain predict overuse low back and lower extremity injury: a prospective cohort study of 545 Navy Cadets	J Sci Med Sport	Excluded	Non RCT	

PACKS-RCT	PMID	Title	Journal/Book	Excluded or Included	Exclusion reason	Randomized ("YES" if selected)
PACK 11	33355211	Differences in sport-related concussion for female and male athletes in comparable collegiate sports: a study from the NCAA-DoD Concussion Assessment, Research and Education (CARE) Consortium	Br J Sports Med	Excluded	Non RCT	
PACK 11	33355155	Time-efficient intervention to improve older adolescents' cardiorespiratory fitness: findings from the 'Burn 2 Learn' cluster randomised controlled trial	Br J Sports Med	Included		YES
PACK 11	33349963	Effect of preconditioning exercise on biceps brachii myotendinous junction displacement during elbow flexor eccentric exercise	Scand J Med Sci Sports	Excluded	Wrong interventio n	
PACK 11	33341995	Nordic Hamstring Exercise training induces improved lower-limb swing phase mechanics and sustained strength	Scand J Med Sci Sports	Excluded	Non RCT	

PACKS-RCT	PMID	Title	Journal/Book	Excluded or Included	Exclusion reason	Randomized ("YES" if selected)
		preservation in sprinters				
PACK 11	33332011	What Role Do Chronic Workloads Play in the Acute to Chronic Workload Ratio? Time to Dismiss ACWR and Its Underlying Theory	Sports Med	Excluded	Non RCT	
PACK 11	33317548	Macroeconomic, demographic and human developmental correlates of physical activity and sitting time among South American adults	Int J Behav Nutr Phys Act	Excluded	Non RCT	
PACK 11	33315813	Blood Glucose Responses during Cardiopulmonary Incremental Exercise Testing in Type 1 Diabetes: A Pooled Analysis	Med Sci Sports Exerc	Excluded	Non RCT	
PACK 11	33315812	Prenatal Exercise and Cardiovascular Health (PEACH) Study: Impact on Muscle Sympathetic Nerve (Re)activity	Med Sci Sports Exerc	Included		YES
PACK 11	33315809	Endurance and Sprint Training Improve Glycemia and VO2peak, but only Frequent Endurance Benefits Blood	Med Sci Sports Exerc	Included		YES

PACKS-RCT	PMID	Title	Journal/Book	Excluded or Included	Exclusion reason	Randomized ("YES" if selected)
		Pressure and Lipidemia				
PACK 11	33315231	Sensitivity and Specificity of Computer-Based Neurocognitive Tests in Sport-Related Concussion: Findings from the NCAA-DoD CARE Consortium	Sports Med	Excluded	Non RCT	
PACK 11	33308235	Combined effects of continuous exercise and intermittent active interruptions to prolonged sitting on postprandial glucose, insulin, and triglycerides in adults with obesity: a randomized crossover trial	Int J Behav Nutr Phys Act	Excluded	Non RCT	
PACK 11	33303369	Outbreak of community-acquired Staphylococcus aureus skin infections in an Australian professional football team	J Sci Med Sport	Excluded	Non RCT	
PACK 11	33298373	Medial tibial stress fracture diagnosis and treatment guidelines	J Sci Med Sport	Excluded	Non RCT	

PACKS-RCT	PMID	Title	Journal/Book	Excluded or Included	Exclusion reason	Randomized ("YES" if selected)
PACK 11	33281095	Acute psycho-physiological responses to perceptually regulated hypoxic and normoxic interval walks in overweight-to-obese adults	J Sci Med Sport	Excluded	Non RCT	
PACK 11	33281094	Calibration of the Active Australia questionnaire and application to a logistic regression model	J Sci Med Sport	Excluded	Non RCT	
PACK 11	33281093	The financial and performance cost of injuries to teams in Australian professional soccer	J Sci Med Sport	Excluded	Non RCT	
PACK 11	33275798	Are adequate PROMs used as outcomes in randomized controlled trials? an analysis of 54 trials	Scand J Med Sci Sports	Excluded	Non RCT	
PACK 11	33273272	The Change in Core Temperature and Sweating Response during Exercise Are Unaffected by Time of Day within the Wake Period	Med Sci Sports Exerc	Excluded	Non RCT	
PACK 11	33270288	Long-term effectiveness of a fundamental motor skill intervention in	Scand J Med Sci Sports	Excluded	Non RCT	

PACKS-RCT	PMID	Title	Journal/Book	Excluded or Included	Exclusion reason	Randomized ("YES" if selected)
		Belgian children: A 6-year follow-up				
PACK 11	33238738	Role of comorbidities in heart failure prognosis Part I: Anaemia, iron deficiency, diabetes, atrial fibrillation	Eur J Prev Cardiol	Excluded	Non RCT	
PACK 11	33108030	One-year aerobic interval training in outpatients with schizophrenia: A randomized controlled trial	Scand J Med Sci Sports	Included		YES
PACK 11	31537125	Risk factors for Lyme carditis: A case-control study	Eur J Prev Cardiol	Excluded	Non RCT	
PACK 12	33355211	Differences in sport-related concussion for female and male athletes in comparable collegiate sports: a study from the NCAA-DoD Concussion Assessment, Research and Education (CARE) Consortium	Br J Sports Med	Excluded	Non RCT	
PACK 12	33361134	Injury patterns differ with age in male youth football: a four-season prospective study of 1111 time-loss	Br J Sports Med	Excluded	Non RCT	

PACKS-RCT	PMID	Title	Journal/Book	Excluded or Included	Exclusion reason	Randomized ("YES" if selected)
		injuries in an elite national academy				
PACK 12	33361135	FIFA Sudden Death Registry (FIFA-SDR): a prospective, observational study of sudden death in worldwide football from 2014 to 2018	Br J Sports Med	Excluded	Non RCT	
PACK 12	33361136	Acute glycaemic management before, during and after exercise for cardiac rehabilitation participants with diabetes mellitus: a joint statement of the British and Canadian Associations of Cardiovascular Prevention and Rehabilitation, the International Council for Cardiovascular Prevention and Rehabilitation and the British Association of Sport and Exercise Sciences	Br J Sports Med	Excluded	Non RCT	

PACKS-RCT	PMID	Title	Journal/Book	Excluded or Included	Exclusion reason	Randomized ("YES" if selected)
PACK 12	33361278	Interassociation consensus recommendations for pitch-side emergency care and personal protective equipment for elite sport during the COVID-19 pandemic	Br J Sports Med	Excluded	Non RCT	
PACK 12	33623999	Prediction of recurrent event in patients with coronary heart disease: the EUROASPIRE Risk Model	Eur J Prev Cardiol	Excluded	Non RCT	
PACK 12	33624001	Safety and feasibility of upper limb cardiopulmonary exercise test in Friedreich ataxia	Eur J Prev Cardiol	Excluded	Non RCT	
PACK 12	33624035	High detection rate and low efficacy in controlling high blood pressure (≥130/80 mmHg) in the community-based health screening: results from KDSAP	Eur J Prev Cardiol	Excluded	Non RCT	
PACK 12	33624071	Combined aerobic/resistance /inspiratory muscle training as the 'optimum' exercise programme for patients with chronic heart	Eur J Prev Cardiol	Included		YES

PACKS-RCT	PMID	Title	Journal/Book	Excluded or Included	Exclusion reason	Randomized ("YES" if selected)
		failure: ARISTOS-HF randomized clinical trial				
PACK 12	33624088	Predicting the transition to and progression of heart failure with preserved ejection fraction: a weighted risk score using bio-humoural, cardiopulmonary, and echocardiographic stress testing	Eur J Prev Cardiol	Excluded	Non RCT	
PACK 12	33624091	Non-exercise estimated cardiorespiratory fitness and mortality from all-causes, cardiovascular disease, and cancer in the NIH-AARP diet and health study	Eur J Prev Cardiol	Excluded	Non RCT	
PACK 12	33624111	Gender gap in risk factor control of coronary patients far from closing: results from the European Society of Cardiology EUROASPIRE V registry	Eur J Prev Cardiol	Excluded	Non RCT	
PACK 12	31537125	Risk factors for Lyme carditis: A case-control study	Eur J Prev Cardiol	Excluded	Non RCT	

PACKS-RCT	PMID	Title	Journal/Book	Excluded or Included	Exclusion reason	Randomized ("YES" if selected)
PACK 12	31311302	Exercise training in cardiovascular disease: are we closing the gender gap?	Eur J Prev Cardiol	Excluded	Non RCT	
PACK 12	31660748	Do angiotensin receptor blockers prevent cardiac events in patients with Marfan syndrome?	Eur J Prev Cardiol	Excluded	Non RCT	
PACK 12	31928362	The 'heart' of preventive cardiology: Lifestyle medicine for the treatment of cardiometabolic diseases	Eur J Prev Cardiol	Excluded	Non RCT	
PACK 12	32320627	Filtered, not unfiltered, coffee in cardiovascular disease	Eur J Prev Cardiol	Excluded	Non RCT	
PACK 12	33308922	The case for promoting physical activity amidst the COVID-19 pandemic	J Sci Med Sport	Excluded	Non RCT	
PACK 12	33341382	Physical activity, mental health and well-being of adults during initial COVID-19 containment strategies: A multi-country cross-sectional analysis	J Sci Med Sport	Excluded	Non RCT	

PACKS-RCT	PMID	Title	Journal/Book	Excluded or Included	Exclusion reason	Randomized ("YES" if selected)
PACK 12	33358586	Trunk endurance, posterior chain flexibility, and previous history of musculoskeletal pain predict overuse low back and lower extremity injury: a prospective cohort study of 545 Navy Cadets	J Sci Med Sport	Excluded	Non RCT	
PACK 12	33376076	Lower preseason reactive strength index scores are associated with injury in female collegiate volleyball players but not male collegiate basketball players	J Sci Med Sport	Excluded	Non RCT	
PACK 12	33402273	Mental health impact on at-risk high-level athletes during COVID-19 lockdown: A pre-, during and post-lockdown longitudinal cohort study of adjustment disorder	J Sci Med Sport	Excluded	Non RCT	
PACK 12	33423947	Eating disorder diagnosis and the female athlete: A longitudinal analysis from college sport to retirement	J Sci Med Sport	Excluded	Non RCT	

PACKS-RCT	PMID	Title	Journal/Book	Excluded or Included	Exclusion reason	Randomized ("YES" if selected)
PACK 12	33315812	Prenatal Exercise and Cardiovascular Health (PEACH) Study: Impact on Muscle Sympathetic Nerve (Re)activity	Med Sci Sports Exerc	Excluded	Non RCT	
PACK 12	33394902	Bone Microarchitecture and Strength Adaptation to Physical Activity: A Within-Subject Controlled, HRpQCT Study	Med Sci Sports Exerc	Excluded	Non RCT	
PACK 12	33433151	Effects of Exercise Mode on Postprandial Metabolism in Humans with Chronic Paraplegia	Med Sci Sports Exerc	Included		YES
PACK 12	33433153	Exercise Reduces Medication for Metabolic Syndrome Management: A Five-Year Follow-up Study	Med Sci Sports Exerc	Excluded	Non RCT	
PACK 12	33270288	Long-term effectiveness of a fundamental motor skill intervention in Belgian children: A 6-year follow-up	Scand J Med Sci Sports	Excluded	Non RCT	
PACK 12	33617077	The burden of injury in field hockey: A secondary analysis of prospective cohort data	Scand J Med Sci Sports	Excluded	Non RCT	
