# Supplementary material for: Methodological rigor and quality of reporting of clinical trials published with physical activity interventions: A report from the Strengthening the Evidence in Exercise Sciences Initiative (SEES Initiative)
Source: PLoS One. 2024 Aug 30;19(8):e0309087. doi: 10.1371/journal.pone.0309087 (PMC11364220; doi:10.1371/journal.pone.0309087)
Supplement: Attachment — Submitted filename: Response to Reviewers.pdf [file pone.0309087.s005.pdf]

Dear Editors,

Thank you for your inquiry regarding the study. I wanted to update you that the Strengthening the Evidence in Exercise Sciences Initiative (SEES Initiative), a two-year project spanning 2019 and 2020, focused on assessing clinical trial quality and has now concluded.

Our primary objective was to consistently share results, providing authors and editors with information to enhance research transparency and methodological rigor. In alignment with our approach to systematic reviews (Oliveira et al, Systematic Reviews 2021), we had planned an extensive publication to showcase the findings of our clinical trial assessments. We believe that PLOS ONE is an ideal journal for disseminating our report.

We appreciate your understanding of these constraints and hope this clarifies the decision to publish results for this specific period.

Dr. Andresa Ignacio
