# Supplementary material for: Methodological rigor and quality of reporting of clinical trials published with physical activity interventions: A report from the Strengthening the Evidence in Exercise Sciences Initiative (SEES Initiative)
Source: PLoS One. 2024 Aug 30;19(8):e0309087. doi: 10.1371/journal.pone.0309087 (PMC11364220; doi:10.1371/journal.pone.0309087)
Supplement: Attachment — Submitted filename: Response to Reviewers (1).pdf [file pone.0309087.s006.pdf]

Dear Editors and Reviewers,

We appreciate the points raised and consider them relevant for enhancing the transparency of our study. Below are our responses to the raised points:

Please ensure that your manuscript meets PLOS ONE's style requirements, including those for file naming:
The manuscript has been reviewed again and adjusted according to PLOS ONE guidelines, as highlighted in the response manuscript.

We note that the grant information you provided in the 'Funding Information' and 'Financial Disclosure' sections do not match:
The funding information has been reviewed and corrected in the submission.

Please include captions for your Supporting Information files at the end of your manuscript, and update any in-text citations to match accordingly:
The supplementary information has been organized and the corresponding captions have been added in the text and at the end of the manuscript.

Please review your reference list to ensure that it is complete and correct. If you have cited papers that have been retracted, please include the rationale for doing so in the manuscript text, or remove these references and replace them with relevant current references. Any changes to the reference list should be mentioned in the rebuttal letter that accompanies your revised manuscript. If you need to cite a retracted article, indicate the article's retracted status in the References list and also include a citation and full reference for the retraction notice:
The reference list has been reviewed, and no changes were necessary.

Reviewer Comments to the Author:

Reviewer #1:
"The manuscript is well written. Objectives and methodology are clearly described. Data analysis is adequate and well performed. The results are relevant for evaluating the quality of Clinical Trials submitted for publication and for producing reliable scientific evidence."

Reviewer #2:

"This work is innovative and relevant to increasing the reliability of the results of articles in the field. The introduction presents the problem clearly, reviewing the state of the art on the subject. The article presents methods and statistics appropriate to the purpose of answering the experimental question. The results have adequate figures and tables; however, the tables could be better designed. The discussion is pertinent and explains the results found, presenting evidence that leads the reader to reflect and be more careful when absorbing the content of RCT-type articles involving physical activity."

Tables 1 and 3 have been reorganized to facilitate visualization. All changes made have been duly highlighted in the article.

Sincerely,
Andresa Ignacio
